# Genetics of Acquired Antibiotic Resistance Genes in *Proteus* spp.

**DOI:** 10.3389/fmicb.2020.00256

**Published:** 2020-02-21

**Authors:** Delphine Girlich, Rémy A. Bonnin, Laurent Dortet, Thierry Naas

**Affiliations:** ^1^EA7361 “Structure, dynamic, function and expression of broad spectrum β-lactamases”, LabEx Lermit, Faculty of Medicine, Université Paris-Saclay, Le Kremlin-Bicêtre, France; ^2^Associated French National Reference Center for Antibiotic Resistance: Carbapenemase-producing Enterobacteriaceae, Le Kremlin-Bicêtre, France; ^3^Evolution and Ecology of Resistance to Antibiotics Unit, Institut Pasteur – APHP – Université Paris-Saclay, Paris, France

**Keywords:** carbapenemase, ESBL, beta-lactamase, urinary tract infections, *Proteus mirabilis*

## Abstract

*Proteus* spp. are commensal Enterobacterales of the human digestive tract. At the same time, *P. mirabilis* is commonly involved in urinary tract infections (UTI). *P. mirabilis* is naturally resistant to several antibiotics including colistin and shows reduced susceptibility to imipenem. However higher levels of resistance to imipenem commonly occur in *P. mirabilis* isolates consecutively to the loss of porins, reduced expression of penicillin binding proteins (PBPs) PBP1a, PBP2, or acquisition of several antibiotic resistance genes, including carbapenemase genes. In addition, resistance to non-β-lactams is also frequently reported including molecules used for treating UTI infections (e.g., fluoroquinolones, nitrofurans). Emergence and spread of multidrug resistant *P. mirabilis* isolates, including those producing ESBLs, AmpC cephalosporinases and carbapenemases, are being more and more frequently reported. This review covers *Proteus* spp. with a focus on the different genetic mechanisms involved in the acquisition of resistance genes to multiple antibiotic classes turning *P. mirabilis* into a dreadful pandrug resistant bacteria and resulting in difficult to treat infections.

## Introduction

**FIGURE 1:**
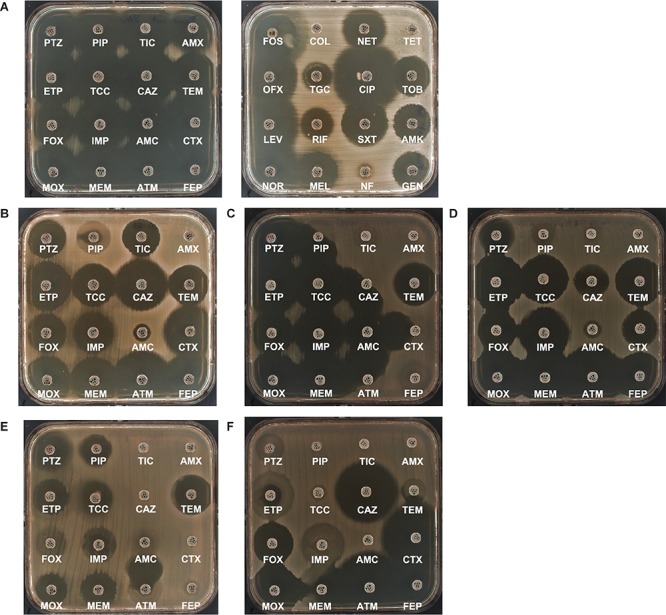
Antibiogram of wild-type *Proteus mirabilis* CIP103181 **(A)**, inhibitors resistant TEM (IRT)- TEM-67-producing *P. mirabilis* NEL (Naas et al., 2003) **(B)**, ESBL producing *P. mirabilis* LIL-1 (Naas et al., 2000) **(C)**, plasmid-encoded cephalosporinase ACC-1 producing *P. mirabilis* SPI (Girlich et al., 2000a) **(D)**, carbapenemase NDM-1 and ESBL VEB-6 producing *P. mirabilis* PEL (Girlich et al., 2015) **(E)**, Carbapenem hydrolyzing oxacillinases (CHDL) OXA-58-producing *P. mirabilis* 1091 (Girlich et al., 2017) **(F)**. β-Lactam abbreviations (from left to right and top to bottom): TZP, piperacillin-tazobactam; PIP, piperacillin; TIC, ticarcillin; AMX, amoxicillin; ETP, ertapenem; TCC, ticarcillin-clavulanic acid; CAZ, ceftazidime; TEM, temocillin; FOX, cefoxitin, IMP, imipenem; AMC, amoxicillin-clavulanic acid; CTX, cefotaxime; MOX, moxalactam; MEM, meropenem; ATM, aztreonam; FEP, cefepime. Non-β-lactam abbreviations (from left to right and from top to bottom): FOS, fosfomycin; COL, colistin; NET, netilmicin; TET, tetracycline, OFX, ofloxacin; TGC, tigecycline; CIP, ciprofloxacin; TOB, tobramicine; LEV, levofloxacin; RIF, rifampicin; SXT, trimethoprim-sulfamethoxazole; AMK, amikacin; NOR, norfloxacin; MEL, Mecillinam; NF, nitrofurantoin, GEN, gentamycin.

*Proteus* spp. are Gram-negative rods belonging to the order of Enterobacterales and the family of *Morganellaceae*. These species are part of the normal bacterial flora of the intestinal tract of humans and animals, and are also widespread in the environment soil and water, where its presence is considered to result from a fecal contamination (O’Hara et al., 2000). *Providencia* spp., and *Morganella* spp., which both formerly belonged to *Proteae* sub-family, have been separated from the *Proteus* genus in 1978, and *Proteus myxofaciens*, never isolated from clinical samples, was proposed to be a member of a new genus, *Cosenzaea* gen. nov. in 2011 (Giammanco et al., 2011). On the basis of their genome-, ribosomal protein- and multi-locus sequence analysis (MLSA)-based phylogenetic trees (Adeolu et al., 2016), the genera *Arsenophonus, Moellerella, Morganella, Photorhabdus, Proteus, Providencia* and *Xenorhabdus* form a distinct, monophyletic grouping, referred to as the *Proteus* – *Xenorhabdus* clade, the type genus being *Morganella*. All these members cluster together in a paraphyletic grouping, sharing seven conserved signature indels (CSIs) in the proteins dihydrolipoamide succinyl transferase, Xaa-Pro dipeptidase, bifunctional UDP-sugar hydrolase (5′-nucleotidase), transcriptional repair coupling factor, phosphate acetyltransferase, histidine–tRNA ligase, and *N*-acetylmuramoyl-l-alanine amidase. These bacteria are oxidase-negative, and negative for arginine decarboxylase and Voges–Proskauer. The main species of the *Proteus* genus is *P. mirabilis*. *Proteus* spp. contains six species: *P. mirabilis, Proteus vulgaris, Proteus penneri, Proteus cibarius, Proteus terrae* and *Proteus hauseri*, and three genomospecies 4, 5, and 6 (O’Hara et al., 2000). *P. mirabilis, P. vulgaris*, and *P. penneri*, are usually described as opportunistic pathogens with a low level of virulence (Giammanco et al., 2011). *P. mirabilis* is the most commonly isolated species from clinical samples, with 90% being from urinary tract infection (UTIs), but also from extra-intestinal infections such as respiratory, eye, ear, nose, skin, burn, meningoencephalitis, osteomyelitis and wound infections (Schaffer and Pearson, 2015). In 1997, *P. mirabilis* was the second most frequently reported enterobacterales (7.7%) in France, after *Escherichia coli* (64.6%) and before *Klebsiella pneumoniae* (5.9%) (de Champs et al., 2000). In Brazil in 2011, *P. mirabilis* was responsible for 13.3% of the infections in intensive care units behind *K. pneumoniae* (56.6%) (Abreu et al., 2011). The genome of *P. mirabilis* contains at least ten adhesion or flagella-mediated motility determinants, involved in both swimming and swarming, which is a central facet of this organism (O’Hara et al., 2000). *P. mirabilis* and *P. vulgaris* are naturally resistant to polymyxins (colistin), nitrofurans, tigecycline and tetracycline (Stock, 2003). *P. vulgaris* produces a chromosomally encoded inducible class A cefuroximase conferring resistance to aminopenicillins, first- and second-generation cephalosporins, with the exception of cefoxitin. *P. mirabilis* does not produce any chromosomally encoded β-lactamase resulting in full susceptibility to all β-lactams for a wild-type phenotype. *Proteus* spp. are usually susceptible to fluoroquinolones. However, strains resistant to antibiotics are increasingly reported, which complicates the treatment of infections caused by *Proteus* spp. Within healthcare facilities, the prevalence of amoxicillin-resistant *P. mirabilis* is close to that of *E. coli* (38% to 48.5%) (Yong et al., 2006) (Figure 1). Extended-spectrum β-lactamases (ESBLs) were first reported in 1983 (Knothe et al., 1983) and plasmid-mediated AmpC β-lactamases were reported in 1988 (Bauernfeind et al., 1989). Typically, ESBLs are mutant, plasmid-mediated β-lactamases derived from older, broad-spectrum β-lactamases (e.g., TEM-1, TEM-2, SHV-1), which have an extended substrate profile allowing hydrolysis of expanded spectrum cephalosporins, penicillins, and aztreonam. These enzymes are most commonly produced by *Klebsiella* spp. and *E. coli* but may also occur in other gram-negative bacteria, including *Morganellaceae*. Currently, *P. mirabilis* isolates have been described with multiple acquired resistance genes encoding narrow spectrum β-lactamases TEM (de Champs et al., 2000), SHV, CARB, IRT (inhibitor-resistant TEM) derivatives (Naas et al., 2003), acquired cephalosporinases [DHA (Bidet et al., 2005) CMY (Decré et al., 2002) ACC-1 (Girlich et al., 2000a)], ESBL type TEM/SHV, CTX-M, VEB (Schultz et al., 2015), PER (Nakama et al., 2016), carbapenemases (Girlich et al., 2015) (Figure 1). Epidemiological studies report a dramatic increase in ESBL producing *P. mirabilis* isolates. Huang et al. (2015) showed that the prevalence of ESBL-producing (mainly CTX-M-14) *P. mirabilis* in Taiwan has increased approximately threefold from 6.2% in 2005 to 20% (28/140) in 2009. In India in 2011, over a 6-month period, 60% of the *P. mirabilis* collected produced an ESBL and among them 19.4% co-produced an AmpC, and 1.7% co-produced a carbapenemase (Datta et al., 2014). A recent study on ceftriaxone non-susceptible Enterobacterales isolates in an University hospital in the United States over a 8-month period in 2015, Tamma et al. (2019) reported that (i) *P. mirabilis* accounted for 7.4% of the Enterobacterales with identified ESBL or AmpC and (ii) the most common ESBL in *P. mirabilis* were CTX-M-1 group, CTX-M-9 group and SHV-type; whereas the most common AmpC was DHA-type (Tamma et al., 2019). In southern Chile at the same period, although the prevalence of CTX-M was high among Enterobacterales, *P. mirabilis* accounted only for 2.2% of them (3/137). These isolates produced simultaneously CTX-M-1-like enzymes, TEM-ESBL (3/3) and SHV-ESBL (2/3) (Pavez et al., 2019). Data from isolates recovered in 2016 and 2017 in Spain from intra-abdominal (IAI) and urinary tract (UTI) infections showed that ESBL producing Enterobacterales were mainly *E. coli* and *K. pneumoniae*. The prevalence of ESBL producing *P. mirabilis* was 0% in IAI and 1.6% in UTI in this study (Cantón et al., 2019).

Prevalence of carbapenemases in *P. mirabilis* is still low, nevertheless, it tends to increase over time worldwide. KPC-2 carbapenemase in *P. mirabilis* was first reported in the United States in 2008 (Tibbetts et al., 2008), and then in a few studies from China 2010 (Sheng et al., 2010) and Brazil 2015 (Cabral et al., 2015). The acquisition of metallo-b-lactamase genes such as *bla*_VIM–like_ genes emerged in Greece in 2006 (Vourli et al., 2006) in Italy in 2008 and more recently in Bulgaria (Markovska et al., 2017). IMP-producing *P. mirabilis* have been identified in only two studies from the United States (Dixon et al., 2016). The presence of *bla*_NDM_ in *Proteus* spp. clinical isolates has been reported in few isolates but in many countries worldwide in New Zealand, in China (*bla*_NDM–__5_), in Bulgaria (*Providencia rettgeri*) in 2016, in Vietnam and in France in 2012 (Girlich et al., 2015). The only descriptions of OXA-48 producing *P. mirabilis* isolates is from Palestine (strains isolated in 2012) (Chen et al., 2015) and from Russia (study reporting data from 2013 to 2014) (Fursova et al., 2015). Some OXA carbapenemases such as OXA-23, or OXA-58-type originating from *Acinetobacter* spp. are now emerging in *P. mirabilis* isolates, as we will see in the present review.

As for ESBL, *ampC* and carbapenemase genes, resistance genes to other antibiotic families such quinolones (*qnr, aac6’Ib*) and to aminoglycosides (APH, AAC, AAD, methylases) are more and more frequently identified in *P. mirabilis*. These genes are carried on mobile genetic elements such as plasmids, transposons, mobile genomic islands, and on integrons that can be mobilized through various mobile elements.

## Genetic Supports of the Resistance in *Proteus* spp.

### Plasmids

Plasmids are the most frequently used vectors for the exchange of resistance genes. They are extrachromosomal, double-stranded, circular DNA molecules capable of autonomous replication and sometimes also transfer (*oriT*). They are host specific or broad-range. Usually, plasmids do not carry genes that are essential for the host survival under “non-stress” conditions and their origin of replication (*ori*) controls their copy number. The diversity of the *ori* genes is commonly used for their classification since two plasmids carrying the same replication origin cannot coexist in the same bacterium and are thus incompatible (Inc group). Carattoli (2009) have developed a molecular typing scheme using PCR amplification to discriminate the different families of plasmids circulating among Enterobacterales, such as IncHI2, HI1, I1-γ, X, L/M, N, FIA, FIB, FIC, W, Y, P, A/C, T, K, B/O. Some of these plasmid families are broad-host range (e.g., IncA/C, IncP, IncQ) that can be stably maintained in many bacterial species (Gram+ and Gram−), while other plasmids have a narrow-spectrum (e.g., IncF, IncI1) (Carattoli, 2009).

### Integrons

**FIGURE 2:**
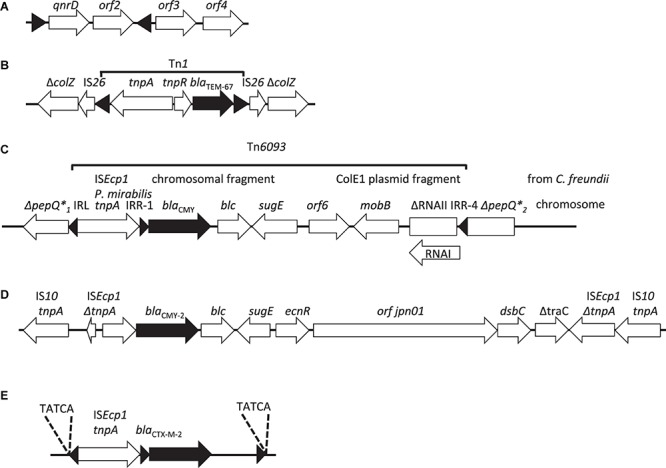
Schematic representation of the structure containing **(A)** the *qnrD* gene in *P. rettgeri* pDIJ09-518a on a non-transmissible plasmid, the right (IRR) and left (IRL) repeated inverted sequences also named “mobile insertion cassette” elements *(mic)*, used for mobilization of *qnrD*, are indicated by black triangles (Guillard et al., 2014); **(B)** the *bla*_TEM–__67_ gene in Tn*1* flanked by two IS*26* on the non-transferable plasmid of *P. mirabilis* NEL (Naas et al., 2003); **(C)** the *bla*_CMY_ gene within the IS*Ecp1*: Tn*6093* transposition module inserted into the *pepQ* gene of *P. mirabilis* (D’Andrea et al., 2006); **(D)** the bla_CMY–__2_ gene inserted into ICE *R391* from *P. mirabilis* PmiJpn1 (Harada et al., 2010); **(E)** the *bla*_CTX–M–__2_ gene located on the chromosomes of *P. mirabilis* TUM4657 (Harada et al., 2012), the 5 letters correspond to the direct repeat sequences (DRL and DRR). The β-lactamase genes and their orientation are represented by black arrows and the IS by black triangles, including the IR-R and IR-L of IS*Ecp1* and the IR-R of the IS*Ecp1* transposition unit.

**FIGURE 3:**
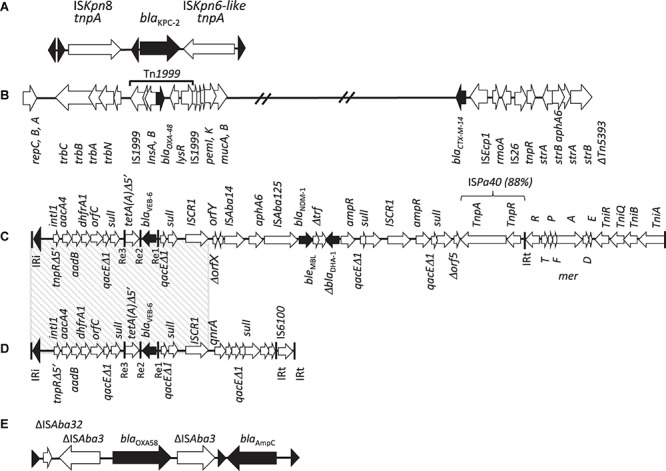
Genetic environments of β-lactamase genes identified in *P. mirabilis*. **(A)** Fragment 3,241 pb from *P. mirabilis*, China, 2015 (Hu et al., 2012), containing *bla*_KPC–__2_, IS*Kpn8* and IS*Kpn6*-like, chimera of several elements of transposons Tn*4401* and Tn*3*, as described for *K. pneumoniae* KP048 (Shen et al., 2009); **(B)** plasmid fragment from OXA-48 and CTX-M-14 -producing *P. mirabilis*, Gaza, Palestine (Chen et al., 2015); **(C)** PGI1-PmPEL genomic island, France, 2016 (Girlich et al., 2015), with the multidrug resistance region (MDR) containing the *bla*_NDM–__1_ and *bla*_VEB–__6_ **(D)** genes and comparison with SGI1-V from *P. mirabilis*, France, 2009 (Siebor and Neuwirth, 2011), containing a 17,1 kb MDR on a complex class 1 integron, including *bla*_VEB–__6_, *aacA4, aadB* and *dhfrA1* genes bracketed by two 3′ conserved segments (3′-CS); **(E)** chromosomal fragment from *P. mirabilis* 1091 isolate, Belgium, 2017 (Girlich et al., 2017), containing *bla*_OXA–__58_ [from *A. baumannii* (Bonnin et al., 2013b)] and *bla*_AmpC_ (from an uncultured bacterium, GenBank N°AMP47568) genes, related to *Acinetobacter bohemicus* (GenBank N° WP_004650432). Triangles represent tandem repeats of 14-pb. The β-lactamase genes and their orientation are represented by black arrows and ISs by black triangles.

Integrons are genetic platforms that allow the incorporation of gene cassettes such as antibiotic resistance genes encoding β-lactamases, aminoglycoside resistance determinants or plasmid-mediated quinolone resistance genes (PMQR). Integrons contain a 5′ conserved segment (5′CS) including an integrase gene (*int*), a 3′CS comprising *qacEΔ1*, a defective *qacE* gene, encoding resistance to biocides and the *sulI* gene for resistance to sulfonamides, an *attI* recombination site, and Pc/P_2_ promoters that allow the transcription of the downstream captured genes. Although integrons are not self-transferable structures, they are most often located on plasmids or transposons, allowing their efficient horizontal gene transfer (Hall and Collis, 1995). More than 130 cassettes of different genes have been identified within integrons bearing resistance to several classes of antibiotics (Partridge et al., 2009). There are complex types of integrons, which in addition to the 5′CS and the 3′CS contain the insertion sequences (ISs), IS*CR1* (previously known as *orf513*) as explained in the following sections, and a partially duplicated 3′CS (Figures 2, 3) (Mokracka et al., 2012).

### Insertion Sequences and Transposons

Bacterial insertion sequences (IS) are small DNA segments (<2.5 kb) with a simple genetic organization. Most IS elements exhibit short terminal inverted repeat sequences (IR) and encode a transposase, an enzyme that is required for transposition. During the process, ISs generally generate a target site duplication (TSD) (Mahillon and Chandler, 1998). These mobile elements play an important role in assembling sets of “accessory” functions in bacteria (such as genes forming parts of degradative or catabolic pathways) and in dissemination of resistance genes. By inserting within a coding-sequence they may inactivate the gene, or by inserting upstream of a gene they may modify its expression. ISs may also help integration of plasmids into the chromosome of bacteria.

Transposons are mobile elements capable of mobilizing foreign DNA through a transposition process. Two main classes of transposons are involved in acquisition of resistance genes:

- Class 1 transposons or composite transposons are made of 2 copies of identical or almost identical ISs that may mobilize the DNA sequence located between them. A known example of this kind of transposon is Tn*1999*, an IS*1999*-composite transposon at the origin of mobilization of *bla*_OXA–__48_ carbapenemase gene from the chromosome of *Shewanella* spp. to an IncL plasmid (Poirel et al., 2012).

- Class 2 transposons or non-composite transposons are made of two IR sequences that surround a transposase gene, transposition accessory genes (such as a replicase, transposition regulatory genes, …) and genes that most often encode resistance determinants. This allows the transposition of the carried DNA with great efficiency. The most widespread β-lactamase, *bla*_TEM–__1_ is carried by a class 2 transposon, Tn*3* (Bailey et al., 2011). The association of integrons with these transferable elements may promote rapid dissemination of antibiotic resistance genes among clinical strains and create further opportunities for inclusion of additional genes within mobile elements.

### Peculiar Insertion Sequences: IS*Ecp1* and IS*CR1*

The mobilization of ESBLs, AmpCs and carbapenemase genes and their exchange between Enterobacterales species and Enterobacterales and non-fermenters may take place using peculiar ISs, such as IS*Ecp1* and IS*CR1*. They are capable of mobilizing nearby DNA, by a mechanism similar to one-ended-transposition. IS*Ecp1* preferentially recognizes flanking sequences as an inverted repeat, rather than its own right IR and thus is capable of mobilizing foreign DNA that is located nearby the right IR (Tamma et al., 2019). Gene mobilization may also take place by “rolling circle” replication *via* specific IS named IS*CR1-* type (or *orf513*) elements. In *P. mirabilis* these types of elements have been described for *bla*_CTX–M–_, plasmid encoded *bla*_a__mpC_, and the *bla*_NDM–__1_ carbapenemase genes in particular (Qin et al., 2015) (Figures 2, 3). The mechanism of mobilization of resistance genes through a rolling circle transposition/recombination process is still not fully understood.

### Integrative Conjugative Elements (ICEs)

Integrative conjugative elements are auto-transferable mobile genetic elements that can be excised from the chromosome *via* their integrase/excisionase gene of the host bacterium and form a circular intermediate that can be transferred by conjugation (Li et al., 2016). The ICE SXT/R391 family is one of the largest ICE families with the most abundant diversity among Gram-negative bacterial species. They carry genes coding for the resistance to sulfamethoxazole, trimethoprim, chloramphenicol, mercury, streptomycin and kanamycin, as well as β-lactamases genes [*bla*_HMS–__1_ (Ryan et al., 2017), *bla*_CMY–__2_ (Harada et al., 2010), *bla*_CTX–M–__2_ (Mata et al., 2011)]. SXT/R391-type ICEs have been identified in strains of *P. mirabilis* not only in Asia (Li et al., 2016) but also in Spain (Mata et al., 2011) and in France (Aberkane et al., 2016). They are easily transferable by conjugation of *P. mirabilis* to *E. coli, K. pneumoniae, Salmonella enterica* serovar *Typhimurium*, or *Citrobacter koseri* (Harada et al., 2010) (Figures 2, 3).

Among ICEs, a specific genomic island described in *Salmonella*, named SGI for *Salmonella* Genomic island (Doublet et al., 2005) have been described in *Proteus* spp. They are integrative elements that can be mobilized by auxiliary plasmids and are classified as IME for Integrative and Mobilizable Elements.

The first SGI1 in *P. mirabilis* was detected from a Palestinian patient in 2006 (Ahmed et al., 2007). It contained genetic determinants conferring resistance to ampicillin, chloramphenicol, streptomycin, sulfonamides, trimethoprim, nalidixic acid and tetracycline. Since then, multiple SGI1 variants have been described in *Salmonella* and are classified from SGI1-A to SGI1-Y, depending on the variable part of their multi-resistance region. Several variants of SGI1, such as SGI1-L and SGI1-U to SGI1-Y have also been described in some *P. mirabilis* isolates (Girlich et al., 2015). Some of these SGIs, integrated in the chromosome of *P. mirabilis*, have recently been renamed PGI (for “*Proteus* Genomic Island”) (Girlich et al., 2015). These pathogenicity islands (PAIs) may also contain multiple β-lactamase-encoding genes, including *bla*_VEB–__6_ (Girlich et al., 2015) and *bla*_NDM–__1_ (Girlich et al., 2015) (Figures 2, 3). Most of these PAIs have lost their horizontal transfer capacity (Schmidt and Hensel, 2004). In *P. mirabilis*, the integration of SGI1/PGI1 into the chromosome always takes place in the 3′ end of the *thdF* gene, encoding a protein involved in oxidation reactions and induced during the stationary phase (Zabel et al., 2000). The strains of *P. mirabilis* carrying these islands have now disseminated also in animals (Schultz et al., 2015).

## Resistance to Aminoglycosides in *Proteus* spp.

In *P. mirabilis*, genetic determinants involved in aminoglycosides resistance are mostly located on integrons as gene cassettes. Most often, these genes are *aadA1*, *aadA2*, coding for aminoglycoside adenyltransferases*; aadB*, coding for aminoglycoside (2′′)-transferase*; aac(6′)-Ib* (also named *aacA4)*, coding for an aminoglycoside acetyltransferase*;* and *sat2*, coding for a streptothricin acetyltransferase (Mokracka et al., 2012). The plasmid-mediated 16S rRNA methylase RmtA was initially characterized in a clinically isolate of *Pseudomonas aeruginosa* (Yokoyama et al., 2003). Then several variants (RmtA, B, C, D and ArmA) have been found in Enterobacterales including *P. mirabilis* (Wachino et al., 2006; Galani et al., 2012; Leulmi et al., 2019). The presence of such determinants on acquired genetic structures also carrying other resistance genes such as the *bla*_NDM_ gene on the plasmid from *K. pneumoniae* Saitama (GenBank AB759690) constitute a risk of co-selection. The acceleration of aminoglycoside resistance among gram-negative bacilli due to plasmid-mediated 16S rRNA methylases, may indeed become an actual clinical hazard in the near future, as the bacteria become resistant to all aminoglycosides.

## Resistance to Quinolones in *Proteus* spp.

It is common to observe in fluoroquinolone-resistant mutants substitutions in the genes encoding chromosomal DNA gyrase (*gyrA* and *gyrB*) or topoisomerase IV (*parC* and *parE*) (Cambau et al., 2006). The rate of resistance related to chromosomal mutations in the fluoroquinolones’ targets was around 70% in Gram-negative enteric isolates in a study by Ogbolu et al. (2011) in Nigeria in 2010. Resistance to quinolones is also mediated by three mechanisms of plasmid resistance: protection of the quinolone target (DNA gyrase and topoisomerase IV) by proteins encoded by the *qnr* genes, acetylation of ciprofloxacin and norfloxacin by the enzyme AAC(6′)-Ib-cr and quinolones specific (QepA) or non-specific (OqxAB) efflux pumps (Zhang et al., 2013). Resistance to quinolones, mediated by *aac(6′)-Ib-cr* and *qnr* was the most frequent, and was observed in 17.2 and 8.2% respectively of the quinolone-resistant strains in the Ogbolu et al. (2011) study, while *qepA* was detected only in 3.2% of these strains. In 2017, this rate was significantly higher in Nigeria where *qnrA* and *aac(6′)-Ib-cr* were detected in, respectively, 36.7 and 5% of the 108 multidrug resistant *P. mirabilis* tested. These genes were most often carried associated with β-lactamase genes (*bla*_TEM_ or *bla*_CTX–M_) on class 1 and class 2 integrons (Alabi et al., 2017). It was observed that the *qnrB10* and *aac(6′)-Ib-cr* genes were significantly associated with the ESBL genes whereas this is not the case for the *qnrB19* nor *qnrD* (Albornoz et al., 2014).

Since, most of the reported *qnrD*-positive isolates belong to *Proteus* or *Providencia* genera, Guillard et al. (2014) hypothesized that *qnrD* originated in *Morganellaceae* before disseminating to other species of Enterobacterales. In these two genera the *qnrD* gene is located on small, non-conjugative 2.7 kb plasmids and ∼4.2 kb mobilizable by “mobile insertion cassette” elements *(mic)* (Guillard et al., 2014) (Figure 2). The *mic* elements are delineated by two IR sequences without transposase gene and a target duplication site, signatures of transposition events (Guillard et al., 2014). *Proteus* spp. harboring plasmids with the *qnrD* genes disseminate worldwide such as, in China (Zhang et al., 2013), Argentina (2014) (Albornoz et al., 2014), Nigeria (Ogbolu et al., 2011), Italy (Mazzariol et al., 2012), France (Guillard et al., 2014) and Poland with *P. vulgaris* (Mokracka et al., 2012). The *qnrC* gene was described in 2009 in China in a strain of *P. mirabilis* and confers only a decrease in susceptibility to ciprofloxacin and levofloxacin. It was located on a 120-kb plasmid co-harboring resistance determinants to ampicillin, sulfamethoxazole, and trimethoprim (Wang et al., 2009). The *qnrA* (Ogbolu et al., 2011) and *qnrS* (Mokracka et al., 2012) genes have been more rarely described in *Proteus* spp. isolates. The *qnrA* genes could have for progenitor an environmental species, *Shewanella algae* (Poirel et al., 2005). In France, it seems that the *qnrA*-containing strains are most often SHV-12 producing *Enterobacter cloacae* and the presence of these genes would be related to the dissemination of an integron of the In*36*-like type (Cambau et al., 2006). In *P. mirabilis*, the presence of *qnrA* remains extremely rare. Only one *qnrA*-producing isolate has been recovered among 1,468 Enterobacterales collected in patients hospitalized in France from 2002 to 2005 (19 *P. mirabilis* in total). This *P. mirabilis* isolate also produced the ESBL PER-1 (Cambau et al., 2006). The *qnrA* gene was located on a 20 to 100 kb plasmid, downstream of IS*CR1* and upstream of *ampR* (encoding the repressor of the *ampC* gene) and *qacE*Δ*1* (a partially deleted gene that would be involved in the resistance to quaternary ammonium compounds). In another strain of *P. mirabilis* isolated in France in 2009, *qnrA* was located on the same genomic island as the *bla_VEB–__6_* gene (SGI1-V) (Figure 2) (Siebor and Neuwirth, 2011). More recently, the *qnrA* gene was identified as chromosomally encoded surrounded by IS*10* and two copies of IS*CR1* in an environmental strain of *P. mirabilis* (Jayol et al., 2016).

## Resistance to β-Lactams in *Proteus* spp.

Ceftazidime, piperacillin-tazobactam, and meropenem are most efficient molecules against *P. mirabilis*, inhibiting > 98.0% of the clinical strains from US hospitals from 2011 to 2013 (Castanheira et al., 2015). However, nearly all the β-lactamases arsenal that has been reported in Enterobacterales has also be described in *Proteus* spp. These β-lactamases ranged from (i) narrow-spectrum β-lactamases with a penicillinase phenotype to (ii) ESBL and acquired AmpCs able to hydrolyze nearly all β-lactams except carbapenems, and finally (iii) carbapenemases of various molecular classes.

### Narrow-Spectrum β-lactamases in *Proteus* spp.

Narrow-spectrum β-lactamases being inhibited by clavulanic acid (e.g., TEM-1 and TEM-2; SHV-1) or being resistant to the action of clavulanic acid (e.g., IRT, OXAs) have been reported in *Proteus* spp. These enzymes hydrolyze narrow-spectrum penicillins, slightly 1st generation cephalosporines (GC) but are not active on 2nd and 3rd GC and carbapenems.

#### TEM-Type

TEM-1 was the first plasmid-mediated penicillinase β-lactamase, described in the 1960s in Gram-negative bacteria. It belongs to the class A or the Ambler classification (Ambler, 1980) and to the group 2b of Bush and Jacoby one (Bush and Jacoby, 2010). Its hydrolytic profile includes penicillins and first-generation cephalosporins (cephalothin). This enzyme is well inhibited by clavulanic acid and tazobactam (Table 1). In *P. mirabilis*, a wide variety of TEM variants have been described including the penicillinases TEM-1 and TEM-2 (Pagani et al., 2003), some TEM resistant to β-lactamase inhibitors such as clavulanic acid, sulbactam and tazobactam [IRT, group 2br (Bush and Jacoby, 2010) including TEM-65, TEM-67, TEM-73, and TEM-74] (de Champs et al., 2000), and finally extended-spectrum TEM enzymes [ESBL, 2be group (Bush and Jacoby, 2010)]. Overall, TEM-1 is the most commonly described enzyme in penicillinase-producing *P. mirabilis* strains. In *P. mirabilis*, IRT enzymes are TEM-2 derivatives, with a cysteine/serine replacing arginine 244 (IRT-1/IRT-2-like). IRT-2 in *P. mirabilis* confers a high level of resistance to amoxicillin and amoxicillin-clavulanate combination but a low level of resistance to ticarcillin (Bret et al., 1996). IRT-2-producing *P. mirabilis* remains susceptible to the combination ticarcillin-clavulanate, piperacillin and cephalothin (Table 1) (Bret et al., 1996). In the study of Bret et al. (1996) the *bla*_IRT–__2_ gene was carried by a conjugative plasmid. In the *P. mirabilis* strain producing the IRT-1 derivative, TEM-67, the gene was carried by a 48-kb non-self-transferable plasmid within a Tn*1*-like transposon (Naas et al., 2003) (Figure 2 and Table 1).

**TABLE 1:** Minimal Inhibitory Concentrations (MIC) of β-lactams in *P. mirabilis* of wild type or β-lactamases producing isolates*^a^*.

	**MIC (mg/L)**													
	**Wild type**	**Penicillinase**	**IRT^b^**	**ESBL**			**Cephalosporinase**			**Carbapenemase**				
**b-lactam**		**TEM-1**	**TEM-67**	**TEM-3**	**TEM-52**	**CTX-M-2**	**CMY-2**	**CMY-3 + TEM-2**	**ACC-1**	**KPC-2**	**NDM-1 + VEB-6**	**OXA-48 + CTX-M-14 + TEM-1**	**OXA-23**	**OXA-58**
AML^b^	1	512	>512	>512	>512	>512	>512	>512	>512	>512	>256		256	>512
AMC	0.5	4	256	4–16	1		>512	>512		>512	64		256	>512
TIC	0.5	128	8	>512	>512			>512	512		256	>128	64	>512
TCC	0.5	2	4	8–16				128	4		32	>2	64	>512
PRL	8	8	4	32–256	>512	>512	>512		128		12		16	>512
TZP	8	0.5		1–2	0.5	0.25	16		8	32	12		0.5	>512
CF		8	16	32		>512		>512	>512				4	ND
FOX					2	0.5	128		4	16			2	
CAZ	0.06		0.5	1–2	32	0.25	16	128	64	16	>256		0.06	0.05
CTX	0.03			2–8	16	>512	8	256		8	>256	>32	0.06	0.03
FEP	ND				64	>512	2		0.25	>16	64	>16		0.09
IPM	0.75			0.5–8	4	0.12	8	4	0.25	8	32	>8	0.25–0.5	0.75
ETP	0.06				0.01					0.25–4	0.25			0.12
MEM	ND				0.25					1–2	0.75		2–4	0.06
DOR												2		
ATM	< 0.03			0.12–0.5	0.5	32		4	0.25		8		0.06	<0.03
TEM	1.5													128

**^a^*Wild type *P. mirabilis* CIP103181 (Girlich et al., 2017), restricted-spectrum penicillinase TEM-1 in *P. mirabilis* CF19 (de Champs et al., 2000); extended-spectrum β-lactamase (BLSE) TEM-3 in *P. mirabilis* PSM1-5 (Mariotte et al., 1994); TEM-52 in *P. mirabilis* DG6499 (Tonkic et al., 2010) and *P. mirabilis* PM685 (Luzzaro et al., 2001); CTX-M-2 in *P. mirabilis* TUM4653 (Harada et al., 2012) and *P. mirabilis* PM15SM02 (Pagani et al., 2003); IRT (TEM-67) in *P. mirabilis* NEL (Naas et al., 2003); plasmid-encoded cephalosporinase CMY-2 in *P. mirabilis* TUM4660 (Harada et al., 2010), CMY-3 (+TEM-2) in *P. mirabilis* CF09 (Bret et al., 1998) and ACC-1 in *P. mirabilis* SPI-1 (Girlich et al., 2000a); carbapenemases KPC-2 in *P. mirabilis* from China (Hu et al., 2012) and Brazil (Cabral et al., 2015)*;* NDM-1 (+VEB-6) in *P. mirabilis* PEL (Girlich et al., 2015); OXA-48 (+CTX-M-14 + TEM-1) in *P. mirabilis* Pm-OXA-48 (Chen et al., 2015); OXA-23 in *P. mirabilis* CF0239 (Bonnet et al., 2002) and OXA-58 in *P. mirabilis* 1091(Girlich et al., 2017). ^b^IRT, Inhibitor-Resistant TEM β-lactamase; AML, amoxicillin; AMC, amoxicillin-clavulanate; ATM, aztreonam; CAZ, ceftazidime; CF, cephalothin; CTX, cefotaxime; DOR, doripenem; ETP, ertapenem; FEP, cefepime; FOX, cefoxitin; IPM, imipenem; MEM, meropenem; PRL, piperacillin; TEM, temocillin; TCC, ticarcillin-clavulanate; TIC, ticarcillin; TZP, piperacillin-tazobactam.*

#### HMS-1

HMS-1 is a β-lactamase that was characterized as IRT in a strain of *P. mirabilis* in 1990 by Thomson et al. (1990). It confers resistance to ticarcillin-clavulanate and a reduced susceptibility to the cefoperazone-sulbactam combination (Thomson et al., 1990). The *bla*_HMS–__1_ gene, located on a SXT/R391 island, has recently been identified by Li et al. (2016) in *P. mirabilis* strains collected from rectal swabs of diarrheal patients and in food (raw meat) from 2008 to 2015 in three cities in China.

#### SHV-Type

SHV-1 is the natural β-lactamase of *K. pneumoniae*, which makes this species also the most prevalent producer of ESBL of SHV-type through point mutation in the active site of this enzyme (Perilli et al., 2002). Few SHV-producing *P. mirabilis* isolates have been reported. They either produce the SHV-1 penicillinase (Chanal et al., 2000) or the SHV-5 ESBL (Ho et al., 2005).

#### CARB/RTG Enzymes

Carbenicillinases of the CARB group were initially reported from *P. aeruginosa* (Labia et al., 1981) then in several species such as *Acinetobacter calcoaceticus* (Choury et al., 2000), *Vibrio cholerae* (Choury et al., 1999) and *P. mirabilis* [RTG-1 (Takahashi et al., 1983), CARB-2/PSE-1 (Chanal et al., 2000)]. These plasmid-encoded enzymes belong to the functional group 2c as they hydrolyze benzylpenicillin, ampicillin, and carbenicillin (Bush and Jacoby, 2010). They are mostly of restricted-spectrum and of low prevalence, especially in Enterobacterales.

#### Narrow Spectrum Oxacillinases

Oxacillinases are enzymes that hydrolyze preferentially cloxacillin and oxacillin and are not or very poorly inhibited by clavulanic acid or tazobactam. In *P. mirabilis* clinical isolates the most prevalent narrow-spectrum oxacillinases are OXA-1, OXA-9, OXA-10 and some point mutant derivatives, such as OXA-320, an OXA-1 single amino-acid mutant that has exclusively been found in *P. mirabilis* from Turkey so far (Cicek et al., 2014). These genes are integron-borne, and thus generally associated with other resistance genes (aminoglycosides, tetracycline, chloramphenicol, etc…), including ESBLs genes such as *bla*_VEB–__1_ (Naas et al., 2000; Kim et al., 2004) even simultaneously associated with carbapenemases genes such as *bla*_VIM–__1_ (Papagiannitsis et al., 2012).

Other ESBLs such as CTX-M-15, SHV-12, TEM-28 or carbapenemases such as NDM-1 have also been described in *P. mirabilis* (Dortet et al., 2012).

### Extended-Spectrum β-Lactamases (ESBLs) in *Proteus* spp.

Although the first reported ESBL were of TEM-type, a diversification of these ESBLs occurred in 2000s with the emergence of CTX-M enzymes, as concomitantly observed in other species of Enterobacterales such as *E. coli* and *K. pneumoniae*. CTX-M enzymes may be divided in five distinct groups of enzymes based on their primary amino acid sequence (Group 1, 2, 8, 9, and 25). Usually, ESBL production provides a high level of resistance to ceftazidime, cefotaxime and aztreonam in *Proteus* spp. The first ESBL-producing *P. mirabilis* isolate has been described in France in 1999 (de Champs et al., 2000). The prevalence of ESBL-producing *Proteus* spp. is 1.3% in Spain, 3.3% in France, 5.9% in Greece (Tonkic et al., 2010), 12.6% in Croatia, 14.5% in Poland and 16.3% in Italy (Nijssen et al., 2004). In Hong Kong, for example, the emergence of ESBL*-*producing *P. mirabilis* strains was monitored in a hospital by Ho et al. (2005) from 1999 to 2002, when the proportion of ESBL-producing *P. mirabilis* increased from 0% in 1999 to 18.5% in 2001 and 25.8% in 2002 (8/31 strains of *P. mirabilis* isolates). The ESBL content of these isolates was more diverse, with CTX-M-13, CTX-M-14, SHV-5 and TEM-11 (Ho et al., 2005). The emergence of CTX-M enzymes was also reported in Japan with CTX-M-2 and CTX-M-10 (Nagano et al., 2003). Jones et al. (2009) reported a prevalence of 7% of ESBL-producers among *P. mirabilis* in Eastern Europe, 10% in Latin America, and 2.2% in North America. In 2013, in a large study including 8,800 isolates of Enterobacterales from the USA, the proportion of ESBL-producers was of 8.4%. In this study, although ESBL-producers were mostly found in *E. coli* (49.5%) and *K. pneumoniae* (40.1%), they represented 5.2% of *P. mirabilis* isolates (Castanheira et al., 2015). In this large collection, Group 1 and Group 9 CTX-Ms were predominant. More sporadically, other rare ESBLs such as PER-1 (Pagani et al., 2003), and VEB (Siebor and Neuwirth, 2011) were detected in *P. mirabilis.* Tonkic et al. (2010) observed that ESBL-producing Enterobacterales were generally susceptible to carbapenems, cefoxitin and to β-lactamase inhibitors, but resistant to most non-β-lactam antibiotic molecules such as ciprofloxacin, trimethoprim/sulfametoxazole, gentamicin, netilmicin, and amikacin. Accordingly, the emergence of *P. mirabilis* strains accumulating resistance mechanisms such as resistance to aminoglycosides (*aacA4, aadB, aphA6*), expanded-spectrum β-lactams (*bla*_VEB–__6_) and carbapenems (*bla*_NDM–__1_) (Girlich et al., 2015) are increasingly reported (Figure 1 and Table 1).

#### TEM-Type and SHV-Type ESBLs

TEM-type ESBLs were identified for the first time in *P. mirabilis* by Mariotte et al. (1994). The *bla*_TEM–__3_ gene was located on a conjugative plasmid of 47-kb (Mariotte et al., 1994). In a French study on >3,000 *P. mirabilis* strains collected between 1997 and 2002, the prevalence of ESBL-producers was 1.3%, all of TEM-type (TEM-24, TEM-52, et TEM-92) (Biendo et al., 2005). Whereas in 2002 in Italy the proportion of strains of ESBL-producing *P. mirabilis* among *P. mirabilis* isolates was 16.3% (98% TEM type), compared with 28.1% for *Providencia stuartii*, 20% for *K. pneumoniae*, and 1.2% for *E. coli* (Spanu et al., 2002).

Several TEM ESBL variants have been identified in *P. mirabilis*, TEM-11 in Hong Kong (Ho et al., 2005), TEM-21 in France (Arpin et al., 2003), TEM-15, -20, -72, -87 in Italy (Perilli et al., 2002), TEM-155 in the US (Perilli et al., 2002). Overall the most prevalent TEM-type ESBL is TEM-52 (Sardelić et al., 2010). In Croatia, the prevalence of ESBL-producing *P. mirabilis* isolates increased from 0.5% in 2005 to 20.9% in 2008, and this ESBL was systematically TEM-52 (Hu et al., 2012). TEM-52-producing *P. mirabilis* strains are susceptible or of reduced susceptibility to cefotaxime and cefepime and are always susceptible to ceftazidime and aztreonam (Pagani et al., 2003) (Table 1). It has been observed that diffusion of TEM-52 producing isolates may be due to the massive use of cefotaxime and moxalactam (Perilli et al., 2002). The *bla*_TEM–__52_ gene is located either on the chromosomal (Tonkic et al., 2010), or on plasmids (Sardelić et al., 2010) whereas *bla*_TEM–__3_, *bla*_TEM–__24_, and *bla*_TEM–__66_ are carried by large plasmids (de Champs et al., 2000). In 2002–2006, TEM-type ESBLs were supplanted by CTX-Ms in 85, 71, and 43% of *E. coli*, *K. pneumoniae*, and *P. mirabilis* positive ESBL strains, respectively (Jones et al., 2009).

As previously mentioned, only few SHV-producing *P. mirabilis* isolates have been reported. Only the SHV-5 ESBL has been described in *P. mirabilis* (Abreu et al., 2011).

#### CTX-Ms

Other class A enzymes with an extended spectrum, CTX-M enzymes are non-TEM, non-SHV ESBLs formerly known as FEC-1 (Matsumoto et al., 1988). Initially reported in the second half of the 1980s, their rate of dissemination has largely increased since 1995 (Bonnet, 2004). The hydrolytic profile includes aminopenicillins (ampicillin, amoxicillin), carboxypenicillins (carbenicillin, ticarcillin), ureidopenicillins (piperacillin), and narrow-spectrum cephalosporins (cephalothin, cephaloridine, cefuroxime), but cefoxitin and carbapenems are spared (Table 1). CTX-M-1 has been initially characterized by a better hydrolysis of cefotaxime than ceftazidime (Bauernfeind et al., 1996a). Currently, there are more than 150 allelic variants of CTX-Ms. These enzymes are classified into five major phylogenic groups: CTX-M-1, CTX-M-2, CTX-M-8, CTX-M-9, and CTX-M-25 (Bonnet, 2004). The worldwide community and nosocomial spread of these enzymes is most commonly associated with *E. coli* ST131 producing CTX-M-15 (Karim et al., 2001). However, in *Proteus* spp., CTX-M-15 is very rarely identified except in a Russian study showing a high prevalence of CTX-M-15 between 2013 and 2014 in >90% of *P. mirabilis* (Fursova et al., 2015). In *Proteus* spp., CTX-M-2, -3, and -14 are the most prevalent variants (Bonnet, 2004). In Japan, CTX-M-2-producing *P. mirabilis* isolates are extremely prevalent (Nakama et al., 2016), but also those producing the variants CTX-M-10 and CTX-M-14 (Nakama et al., 2016). The prevalence of ESBL-producing *P. mirabilis* isolates has increased steadily in Japan to a dramatically high level from 2000 to 2004 (46.2%) (Nakano et al., 2012), much higher than in France (6.9%) (Chanal et al., 2000) or in the United States (9.5%) (Saurina et al., 2000). Nakama et al. (2016) recently reported that the prevalence of CTX-M-2 and CTX-M-14-producing *P. mirabilis* in a Japanese hospital between 2013 and 2014 was 11.1% of the *P. mirabilis* isolates versus 11.5% of *E. coli*, and 6.2% of *K. pneumoniae* isolates. CTX-M-2 ESBLs have also been reported in *P. mirabilis* isolates in Spain (Mata et al., 2011), Argentina (Quinteros et al., 2003), and Italy (Pagani et al., 2003).

Nakano et al. (2012) showed the presence of the *bla*_CTX–M–__2_ gene on plasmids of different incompatibility groups, mostly IncT, but also IncW, IncK, IncHI1, IncX, IncN, and IncW. In all isolates, IS*Ecp1* was identified 49-bp upstream of the *bla*_CTX–M–__2_ gene, likely responsible of its gene mobilization and bringing the −10 and −35 promoter sequences for its expression (Nakano et al., 2012). Harada et al. (2012) showed the simultaneous localization of the *bla*_CTX–M–__2_ gene on a incT plasmids and on the chromosome of a *P. mirabilis* isolate. The integration of *bla*_CTX–M_ genes in the chromosome is mediated by transposition via the upstream-located IS*Ecp1* insertion sequence (Figure 2) (Harada et al., 2012). IS*Ecp1* has also been involved in the mobilization of the *bla*_CTX–M–__14_ gene in Gaza (Palestine) (Chen et al., 2015). Among the other CTX-M variants, CTX-M-3-producing *P. mirabilis* were reported in France (Lartigue et al., 2005), CTX-M-8 producers in Brazil (Bonnet et al., 2000), and CTX-M-14 and CTX-M-27 producers in the United States (Castanheira et al., 2015), and Korea (Yong et al., 2006). Although, in the United States, CTX-M-14 and CTX-M-27 have been more often reported in *E. coli*, they have been described also in *K. pneumoniae* and *P. mirabilis* (Castanheira et al., 2015). In Korea, CTX-M-14 was first identified in *K. pneumoniae*, *E. coli, Shigella sonnei*, and then in *P. mirabilis* between 2002 and 2003 (Yong et al., 2006). More sporadically, CTX-M-8/-25 variants in the USA (Castanheira et al., 2015), CTX-M-24 in Vietnam between 2009 and 2011 (Biedenbach et al., 2014), have been reported in *P. mirabilis*. All these studies suggest that CTX-Ms are gradually spreading among *P. mirabilis* isolates as for other Enterobacterales. More worryingly, two studies showed the simultaneous diffusion of carbapenemase genes (*bla*_IMP–__6_) with *bla*_CTX–M–__2_ in Japan (Ohno et al., 2017) and *bla*_OXA–__48_ with *bla*_CTX–M–__14_ in Palestine (Chen et al., 2015) (Figure 3).

ESBLs are commonly found in human clinical isolates of *P. mirabilis*, but more rarely from strains of animal origin. The first CTX-M-producing *P. mirabilis* CTX-M-55, was isolated in 2013 from a macaque imported from Vietnam to France in 2011 (Dahmen et al., 2013). Within veterinary strains of *Morganellaceae*, *bla*_CTX–M–__15_ (*P. mirabilis*) and *bla*_CTX–M–__1_ (*P. rettgeri*) have been recently reported. In this study, the *bla*_CTX–M–__15_ gene was even detected in duplicate, one chromosomally- and the other plasmid-located (non-typable) (Schultz et al., 2017).

#### VEB Enzymes

VEB, or “Vietnamese ESBL,” is an ESBL initially identified in France in a *E. coli* strain from a Vietnamese patient in 1999 (Poirel et al., 1999). The *bla*_VEB–__1_ gene has been reported in several non-fermenting Gram-negative Enterobacterales and Gram-negative rods in Asia, Europe, Africa and America, located either on the chromosome or on plasmids, in a gene cassette in a class 1 integron together with gene cassettes *bla*_OXA–__10_, *arr-2*, and *aadB* (Poirel et al., 1999), however, the cassette array may contains other cassettes (Kim et al., 2004).

The antibiotic susceptibility panel of VEB-1-producing *P. mirabilis* strains shows an unusual synergy between cefoxitin and cefuroxime (Naas et al., 2000). In *P. mirabilis*, the *bla*_VEB–__1_ gene was first described in Korea (Kim et al., 2004), on a non-transferable genetic support and in Greece in 2010 (Papagiannitsis et al., 2012) on a conjugative 120 kb plasmid (IncA/C2) carrying also *bla*_VIM–__1_, *bla*_OXA–__10_ and *bla*_TEM–__1_ genes. The *bla*_VEB–__1_ gene was located within a class 1 integron preceded by IS*1999* and with gene cassettes *aadB, arr2, cmlA5, bla*_OXA–__10_, and *aadA1*, a truncated 3′CS at the *sulI* gene, and having integrated additional *cmlA9-tetR* (G) -*tetA* (G) genes associated with the IS*CR6* insertion sequence (Papagiannitsis et al., 2012). On the same plasmid, the *bla*_VIM–__1_ gene, with the gene cassettes *aacA7, dfrA1*, and *aadA1*, were included in an integron with a 5′CS fragment interrupted by the IS*26*-Δ*orf6*-IS*6100* sequence. It seems that the plasmid acquired by this *P. mirabilis* isolate underwent several recombination events between different structures (Papagiannitsis et al., 2012). Several VEB-6-producing *P. mirabilis* isolates have been described later in several infections in France (Siebor and Neuwirth, 2011), in Oman (Potron et al., 2009), in Australia (Zong et al., 2009). VEB-6-producing strains have also been isolated from animal samples (Schultz et al., 2015) and raw meat in Switzerland (Seiffert et al., 2013). In this latter study, the *bla*_VEB–__6_ gene was located in a class I integron of ∼17-kb also carrying multiple resistance genes such as *aacA4, aadB, dfrA1, sul1, tet(A)*, and *qnrA1* (Nordmann and Naas, 1994). The *bla*_VEB–__6_ gene has been recurrently reported on a SGI1-V genetic islands also carrying the *qnrA1* gene in a *P. mirabilis* isolate recovered from a blood culture in France in 2009 and in veterinary strains (Schultz et al., 2015)^.^ In France in 2012, the *bla*_VEB–__6_ gene was reported together with the NDM-1 carbapenemase encoding gene in a novel island called “*Proteus* genomic island, PGI1-PmPEL” in a *P. mirabilis* strain isolated from the urine of a patient previously hospitalized in Switzerland (Girlich et al., 2015) (Figures 1, 3 and Table 1).

#### PER Enzymes

The PER-1 ESBL was firstly identified in France in a *P. aeruginosa* isolate from a patient originating from Turkey (Nordmann and Naas, 1994), where this enzyme was shown to spread widely among non-fermenting Gram-negative rods (Bahar et al., 2004). This enzyme is very different in structure and function from other ESBLs (Philippon et al., 2016) and confers resistance to penicillins, cefotaxime, ceftazidime, and aztreonam. The presence of PER-1 in *P. mirabilis* is anecdotal: one strain has been described in Spain [among fecal Enterobacterales (Philippon et al., 2016)], another was responsible for an outbreak in one hospital in Italy (Pagani et al., 2003). The success of the epidemic PER-1-producing *P. mirabilis* isolate in the study by Pagani et al. (2003) in northern Italy might be due, at least in part, to the high levels of resistance reached by the strain, which also produce TEM-2.

The PER-2 variant, sharing 86% amino acid identity with PER-1, was detected only in Enterobacterales *(E. coli, K. pneumoniae, P. mirabilis, S. typhimurium)*. The spread of PER-2 remains limited to South America (Bauernfeind et al., 1996b). Upstream of the *bla*_PER–__2_ gene, an insertion sequence IS*Pa12* containing the gene encoding a transposase has been identified. IS*Pa12*, or IS*1387a*, systematically associated with the *bla*_PER_ gene on plasmids or chromosomes, would be involved in the mobilization and expression of PER-1 and PER-2 (Power et al., 2007).

### Plasmid-Encoded Cephalosporinases (AmpC)

In the late 1980s, cephalosporinase (AmpC) genes of chromosomal origin (*Citrobacter freundii, E. cloacae, Morganella morganii, Hafnia alvei*) were identified on plasmids spreading among Enterobacterales such as: *Klebsiella* spp., *E. coli*, *P. mirabilis*, and *Salmonella* spp. (Jacoby, 2009). AmpC are class C β-lactamases that belong to the functional group 1 of Bush and Jacoby (Bush and Jacoby, 2010). These enzymes are characterized by hydrolytic activities toward cephamycins (except ACC-1) (Girlich et al., 2000a) and other expanded-spectrum cephalosporins (e.g., cefotaxime, ceftazidime), except cefepime and carbapenems. They are not inhibited by clavulanate and tazobactam but by cloxacillin (Table 1). Most plasmid-encoded AmpCs are constitutively expressed except inducible DHA-1, ACT-1, and CFE-1 (Gaillot et al., 1997). Acquired cephalosporinases in *Proteus* spp. confer a high-level of resistance to amoxicillin, ticarcillin, cefoxitin, cefotaxime and ceftazidime and a decreased susceptibility to aztreonam. The prevalence of plasmid-mediated cephalosporinases among Enterobacterales varies according to various studies, e.g., from 0.06% in 1999 to 1.3% in 2007 in Spain (Mata et al., 2010). In the study from Moland et al. (2006) about Enterobacterales isolates collected in American hospitals between 2001 and 2002, the prevalence of plasmid-mediated AmpCs was higher in *K. pneumoniae* (3.6%) than in *P. mirabilis* (1.4%). The situation was different in Korea in 2008, where the highest prevalence of plasmid-mediated AmpCs was in *P. mirabilis* (3.6%), although it remained low as compared to that of the ESBLs (12.6% of *P. mirabili*s isolates) (Song et al., 2011). In this study, the identified plasmid-mediated cephalosporinases were DHA (Bidet et al., 2005), CMY (Decré et al., 2002), FOX-5 (Moland et al., 2006), and ACC-1 (Girlich et al., 2000a). A more recent large study on resistance to ceftazidime-avibactam in the United States showed that, conversely to other Enterobacterales that produce ESBLs, *P. mirabilis* most often carried plasmid-encoded AmpC (pAmpC) (66.7%) (Mendes et al., 2019).

#### CMY Enzymes

CMY-2 (or BIL-1, or LAT-2, originating from *C. freundii*), and its derivatives, are the most common plasmid-mediated cephalosporinases in *P. mirabilis* (Mata et al., 2011). A study on AmpC-producing *P. mirabilis* collected in different European countries from 1999 to 2008 showed the high prevalence of chromosomally located *bla*_CMY–__2_ genes (D’Andrea et al., 2011). The *bla*_CMY_ gene may also be located on plasmids of IncA/C or IncI1 incompatibility group (Carattoli, 2009). IS*Ecp1* has been frequently described upstream of the *bla*_CMY–__2__–like_ genes along with other IS, such as IS*5*, IS*10*, or IS*1294*, that are likely involved in the mobilization of these genes (Verdet et al., 2009). In the transposition module called Tn*6093*, IS*Ecp1* is located 110-bp upstream of the *bla*_CMY_ gene, and contains fragments of *C. freundii* chromosome on a *ColE1-*type plasmid (D’Andrea et al., 2011) (Figure 2). Mobilization of the *bla*_CMY–__2_ gene by integrative and conjugative islands (ICE) has also been described in Asia (Li et al., 2016), Spain (Mata et al., 2011), and France (Aberkane et al., 2016), in *Proteus* isolates from humans (Harada et al., 2010) or from livestock, wild animals and pets. The *bla*_CMY–__2_ gene has also been described in Switzerland from chicken raw meat on a IncI1 plasmid (Seiffert et al., 2013). Recently, a study by Schultz et al. (2017) showed the chromosomal localization of *bla*_CMY–__2_ and *bla*_DHA–__16_ genes in *Morganellaceae* isolates collected from pets. The distribution of CMY variants differs from country to country: *bla*_CMY–__4_, *bla*_CMY–__12_, *bla*_CMY–__14_, *bla*_CMY–__15_, *bla*_CMY–__38_, and *bla*_CMY–__45_ are the most prevalent in Poland, *bla*_CMY–__16_ in Greece and in Italy (D’Andrea et al., 2011). A strain of *P. mirabilis* producing CMY-42, a CMY-2 variant, was collected in Egypt (Helmy and Wasfi, 2014). Other point-mutation variants of CMY-2 have been identified in *P. mirabilis* likely inserted into the chromosome. This is the case for CMY-3 (Bret et al., 1998) in France; CMY-4, -12, -14, and -15 in Poland from 1999 to 2001 (Literacka et al., 2004), in Tunisia in 1998 (Verdet et al., 1998), and in France in 2002 (Decré et al., 2002); CMY-15 in *P. mirabilis* and *P. vulgaris* (Mokracka et al., 2012); and CMY-16 identified in Italy in 2003 (D’Andrea et al., 2006).

#### DHA-1

DHA-1 is the inducible chromosome-encoded cephalosporinase from *M. morganii* (Verdet et al., 2000). The *bla*_DHA–__1_ gene was firstly detected plasmid-encoded in a *Salmonella enterica* isolate from Saudi Arabia in 1997 (Gaillot et al., 1997). Then, dissemination of DHA-1-producing *K. pneumoniae* has been reported in Taiwan in 2002 (Yan et al., 2002). This plasmid-acquired cephalosporinase has been reported for the first time in a *P. mirabilis* isolate in 2005 in France (Bidet et al., 2005) and in Korea (Yong et al., 2005). Yong et al. (2005) showed the high prevalence of DHA-1-producing Enterobacterales in one hospital in Korea. In this hospital, among the cefoxitin resistant isolates 32% were *K. oxytoca*, 21.1% were *K. pneumoniae*, 8.3% were *P. mirabilis* and 2.7% were *E. coli.* All producing the DHA-1 enzyme (Yong et al., 2005). Usually, the *bla*_DHA–__1_ gene is located downstream of its *ampR* regulatory gene, divergently transcribed, and with overlapping promoters, on conjugative plasmids. They were mobilized and inserted into a complex integron, probably as a result of integrase-mediated recombination (Verdet et al., 2000). Such events have also led to the integration of a fragment containing *bla*_DHA–__1_ gene and its *ampR* regulatory gene downstream of *bla*_NDM–__1_ into the PGI-*Pm*PEL, also carrying *bla*_VEB–__6_ gene in a strain of *P. mirabilis* described in France in 2015 (Girlich et al., 2015) (Figure 3). DHA-1 has also been identified sporadically in *P. vulgaris* in Poland in 2012 (Mokracka et al., 2012).

#### FOX Enzymes

The FOX family is a distinct family of AmpCs in terms of protein sequence identity (Papp-Wallace et al., 2014). The substrate specificity of this class C β-lactamase includes cephamycins. The *bla*_FOX_ gene is widely disseminated in the United States on IncA/C and pMG252 plasmids and is often associated with the plasmid mediated quinolone resistance gene *qnr* (Papp-Wallace et al., 2014). FOX β-lactamases have also been described in Europe (Bauernfeind et al., 1997), South America (Gonzalez Leiza et al., 1994), United Kingdom and Ireland (Manoharan et al., 2012) in *E. coli, Enterobacter* spp. and *Klebsiella* spp. isolates. FOX-5-producing *P. mirabilis* isolates have been detected only once in the United States in 2002 (Moland et al., 2006).

#### ACC-1

ACC-1 is another family of plasmid-encoded cephalosporinase originating from the Enterobacterales species *H. alvei* (Girlich et al., 2000b). ACC-1 was detected simultaneously in strains of *E. coli* and *P. mirabilis* in the same patient in France (Girlich et al., 2000a). *K. pneumoniae, Salmonella* spp. and *P. mirabilis* isolates producing ACC-1 have been collected in several wards of a Tunisian hospital (Rhimi-Mahjoubi et al., 2002) and in Spain (Mata et al., 2010). Production of ACC-1 is particularly suspected in isolates that are resistant to ceftazidime, without any synergy in the presence of clavulanate, together with full susceptibility to cefoxitin (in fact cefoxitin behaves as an inhibitor) and of decreased susceptibility to cefpirome (Girlich et al., 2000a) (Figure 1). Of note, the production of ACC-1 can be masked by the production of an ESBL. The 112-kb plasmids identified in *P. mirabilis* and *E. coli* carrying *bla*_ACC–__1_ did not carry the *ampR* gene upstream of *bla*_ACC–__1_ and the production of this cephalosporinase was constitutive (Girlich et al., 2000a).

### Carbapenemases in *Proteus* spp.

Carbapenems (imipenem, ertapenem, meropenem, doripenem) remain in many countries the “last resort” antibiotics for the treatment of severe infection caused by ESBL-producing Enterobacterales. The recent emergence and rapid spread of carbapenemase-producing Enterobacterales (CPE) is a major public health issue as clinical therapeutic options are considerably limited. Invasive infections caused by CPE are often linked to a high mortality rate. Acquired carbapenemases identified in *Proteus* spp. are (i) Ambler class A carbapenemases such as KPC-2, (ii) metallo-β-lactamases (MBLs, Ambler class B) VIM-1, IMP-type and NDM-1, and (iii) carbapenem-hydrolyzing Ambler class D β-lactamases (CHDLs) such as OXA-48 or more intriguingly OXA-23, OXA-40 and OXA-58 (carbapenemases specifically identified in *Acinetobacter* spp.). In Europe, the most prevalent carbapenemases are KPC, NDM, VIM and OXA-48. In United States and in Japan, the most prevalent carbapenemase is KPC and IMP respectively (Naas et al., 2008; Ohno et al., 2017).

The detection of CPEs is essential in the control of infections and can have an impact on the treatment. Unfortunately, this detection is particularly difficult in *Proteus* spp. in which the level of resistance to carbapenems remains low despite the production of a carbapenemase (Girlich et al., 2017). In addition, the “swarming” of *Proteus* spp. does not allow the efficient detection of CPE using phenotypic inhibition methods with disks containing dipicolinic acid (or ethylene diamine tetraacetic acid, EDTA), boronic acid and temocillin (van Dijk et al., 2014). On top of that, *Morganellaceae* (particularly *P. mirabilis*) possess intrinsic decreased susceptibility to imipenem (but not meropenem and ertapenem), mainly due to penicillin binding proteins (PBPs) with weak affinity or porin loss. Consequently, ertapenem and/or meropenem have to be used to screen carbapenemase producers in *Morganellaceae*.

#### Ambler Class A Carbapenemase: KPC-2

Initially identified in a *K. pneumoniae* isolate from the US (Yigit et al., 2001), KPC-2 and variants went global, becoming the most common carbapenemase that has disseminated widely among Enterobacterales (Naas et al., 2008) *P. aeruginosa* (Wolter et al., 2009) and more rarely in *A. baumannii* (Robledo et al., 2010). It confers resistance to all β-lactams, including carbapenems, cephalosporins, cephamycins, penicillins and monobactams. The *bla*_KPC_ genes are most commonly located on transferable plasmids of different sizes and structures, within transposons of Tn*3* type, Tn*4401* (Naas et al., 2008). Tn*4401*, 10 kb long, is bracketed by two imperfect repeats of 39-bp. It contains the genes encoding a transposase and a resolvase and two ISs, IS*Kpn6* and IS*Kpn7* (Naas et al., 2008) or IS*Kpn6*-like and IS*Kpn8* (Shen et al., 2009). In *P. mirabilis*, KPC-2 was identified for the first time in 2008 in the blood culture of a diabetic patient in the United States (Tibbetts et al., 2008). Then, KPC-2 producing *P. mirabilis* isolates have been identified in China (Shen et al., 2009; Sheng et al., 2010; Hu et al., 2012), Colombia (Cuzon et al., 2011)Brazil (Cabral et al., 2015), and Italy (Di Pilato et al., 2016). The *bla*_KPC–__2_ gene was located on 45–54 kb conjugative plasmids, surrounded by IS*Kpn8* and IS*Kpn6*-like, as the structure described in *K. pneumoniae* KP048 (Shen et al., 2009) (Figure 3). In Brazil, the identification of KPC-2 at the origin of carbapenem resistance in *P. mirabilis* is worrying as it results pandrug resistant isolates. Indeed *P. mirabilis* is naturally resistant to polymyxins, one of the only drugs showing some efficacy against KPC-producers. However, this discovery is not entirely unexpected, given the number of publications reporting the spread of the *bla*_KPC_ genes between different Gram-negative bacterial species such as *K. pneumoniae* and *P. aeruginosa* in the same hospital (Recife, Brazil) (Cabral et al., 2015).

#### Metallo-β-Lactamases

The MBLs are structural Ambler class B β-lactamases and functional group 3 enzymes (Bush and Jacoby, 2010). MBLs hydrolyze all β-lactams except aztreonam. MBLs have one or two divalent zinc ions in their active site and are inhibited *in vitro* by cation chelators such as EDTA. MBL are classified into 3 subclasses according to their protein sequence: B1, B2, and B3. Class B1-like and plasmid-encoded enzymes such as NDM, VIM, and IMP have emerged in clinical settings in both Enterobacterales and non-fermenting Gram-negative bacilli.

##### IMP

IMP carbapenemase for “imipenemase” is the most common carbapenemase in Enterobacterales in Japan (Ohno et al., 2017). IMP-1 was originally described in a strain of *P. aeruginosa* in Japan in 1991 (Watanabe et al., 1991). Then, also in Japan, the *bla*_IMP–__1_ gene has been described to be located on a IncP-9 conjugative plasmid, in a *Serratia marcescens* isolate (Osano et al., 1994). The *bla*_IMP_ gene is, most of the time, located on a broad host range plasmid, such as IncL/M and IncA/C. On these plasmids, the *bla*_IMP_ gene is most often part of a class 1 integron as a gene cassette, along with other resistance determinants encoding aminoglycoside acetyltransferase (e.g., *aacA4*), aminoglycoside adenilyltransferase (*aad1*, *aad2*) or narrow-spectrum oxacillinases (e.g., *bla*_OXA–__1__,–__2__,–__10_). Ohno et al. showed the steady increase in the prevalence of CPE from 2010 to 2013 in a Japanese hospital; from 0.1% in 2010, 0.2% in 2011, 0.2% in 2012 to 0.8% in 2013 (Ohno et al., 2017). In this study, all of these strains (17 out of 4875 isolates) produced IMP-6 along with the ESBL CTX-M-2 for most of them (Ohno et al., 2017). In addition, a multi-center study on metallo-β-lactamase producing Enterobacterales (MBL) showed a global CPE prevalence of 0.42% in a Kinki hospital in Japan between 2000 and 2002 comprising 96 IMP-1, IMP-2 and VIM-2, positive isolates but no *Proteus* spp. (Nishio et al., 2004). The acquisition of *bla*_IMP_-like genes by *P. mirabilis* seems to be a rare event, since only two descriptions of IMP-27 carried by a class 2 integron has been reported to date (Dixon et al., 2016). These isolates were collected in 2009 and 2015 from two different patients who were from two different states in the upper plain region of the United States. An IMP-27-producing *P. mirabilis* was also identified in the United States in a soil sample in a pig farm in 2015 (Mollenkopf et al., 2017). The *bla*_IMP–__27_ gene was located on an IncQ1 plasmid. It has been suggested in this study that the dissemination of this plasmid among Enterobacterales could be facilitated by the use of ceftiofur in pig farms in the United States (Mollenkopf et al., 2017).

A more recent study by Ramos et al. (2017) reports an outbreak of 10 IMP-1 producing *P. mirabilis* isolates (two distinct clones) in a tertiary hospital in São Paulo, Brazil, in 2015. In this study the *bla*_IMP–__1_ gene was carried on a novel class 1 integron, In*1359*, on a conjugative plasmid belonging to the IncA/C group. More worrisome was the coproduction of additional plasmid-mediated resistance genes *bla*_KPC–__2_, *bla*_CTX–M–__14_ and *rmtB*-1.

##### VIM-1

VIM-1 belongs to a second major group of class B1 MBLs. It was first described in 1997 in Italy, hence its name: “Verona integron-encoded MBL” in a clinical strain of *P. aeruginosa* (Lauretti et al., 1999). VIM-type enzymes are predominant in *P. aeruginosa* isolates, but they may also be found in Enterobacterales, especially in Mediterranean countries such as Italy, Greece and Tunisia (Mojica et al., 2016). The *bla*_VIM_ genes are generally inserted into class 1 integrons that are themselves located on plasmids of IncN or IncFI/FII groups, or integrated into the chromosome (Mojica et al., 2016). More than 66 variants have been described (Naas et al., 2017) (BLDB^1^, last accessed 12/12/19) that can be divided in three distinct sub-families VIM-1-like (31 variants), VIM-2-like (33 variants; 90% AA sequence identity with VIM-1) and VIM-7-like (2 variants; 80% AA sequence identity with VIM-1).

In *P. mirabilis*, the prevalence of VIM-1 is especially high in Greece, where the first strain was described in 2006 (Papagiannitsis et al., 2012) and where the spread has been described not only in hospital settings, but also in the community (Tsakris et al., 2007). The *bla*_VIM–__1_ gene is located on class 1 integrons, inserted into the chromosome with genes *aacA7, dhfr*, and *aadA* (Vourli et al., 2006) or in conjugative plasmids carrying also *bla*_VEB–__1_, *bla*_OXA–__10_, *bla*_TEM–__1_ genes (Papagiannitsis et al., 2012). In addition, an outbreak of 13 *P. mirabilis* isolates producing VIM-1 and CMY-99 was recently reported in Bulgaria (Markovska et al., 2017). In this clone, the *bla*_VIM–__1_ gene was located into a class 1 integron that also contained the aminoglycoside (*aac(6′)-Ib, ant(3″)-Ia*) and trimethoprim (*dhfrA1*) resistance determinants (Markovska et al., 2017) Two other isolates of *P. mirabilis* co-producing VIM-1 and CMY-16 have also been described in the Netherlands (van Dijk et al., 2014).

##### NDM-1

The “New Delhi metallo-β-lactamase-1” (NDM-1) was initially identified in 2008 in a *K. pneumoniae* isolate recovered from a patient repatriated from India. As other MBLs, this enzyme confers resistance to carbapenems and all β-lactams with the exception of aztreonam (Yong et al., 2009). Endemic in the India sub-continent, the *bla*_NDM–__1_ gene has spread throughout the world, not only in Enterobacterales, but also in various species such as *Vibrio cholerae*, *Pseudomonas* spp. and *Acinetobacter* spp. In Enterobacterales, the *bla*_NDM–__1_ gene is rarely located on the chromosome, but most often on conjugative plasmids from different incompatibility groups (Poirel et al., 2011) such as IncA/C, IncF, IncL/M, IncHI1B or more recently IncX3 of ∼40 kb in size (Zhang et al., 2016). The immediate genetic environment of *bla*_NDM_ is characterized by an upstream inserted IS*Aba125* that originated from *A. baumannii* (Poirel et al., 2011) and a downstream-located *ble*_MBL_ gene, encoding a bleomycin binding protein, BRP_MBL_ (Dortet et al., 2017). It has recently been evidenced an association of *bla*_NDM_ gene with IS*CR1* insertion sequence, one of the most widespread mechanisms for the diffusion of clinically relevant antibiotic resistance genes (Liu et al., 2013). The presence of *bla*_NDM_ in *Proteus* spp. clinical isolates is still episodic in New Zealand (Williamson et al., 2012), in China (*bla*_NDM–__5_) (Zhang et al., 2016), in Bulgaria (*P. rettgeri*) in 2016 (Pfeifer et al., 2017), in Vietnam (Tran et al., 2015). However, the *bla*_NDM–__1_ gene was the first carbapenemase gene identified on a genomic island (PGI-1, PGI1-PmPEL) together with the *bla*_VEB–__6_ gene in *P. mirabilis* isolate recovered from the urine of an hospitalized patient in France in 2012 (Girlich et al., 2015) (Figures 1, 3). Surprisingly, in *P. mirabilis*, the level of resistance to carbapenems conferred by NDM-1 remains very low; with the exception of imipenem (MIC 32 μg/mL). Indeed, the MIC of ertapenem (0.25 μg/mL) and meropenem (0.75 μg/mL) remain within the sensitivity range defined by the Clinical and Laboratory Standards Institute (CLSI) (Table 1). More recently, Baraniak et al. (2016) also identified *bla*_NDM–__1_ and *bla*_VEB–__6_ genes on the chromosome of a *P. mirabilis* isolate in a patient from Afghanistan on a Tn*125* transposon.

The *bla*_NDM–__1_ gene was also identified in China on a 58-kb conjugative plasmid inserted within a class 1 integron downstream of IS*CR1* (*qacE*Δ*1/sul1-*IS*CR1-1-trpF-ble*_MBL_*-bla*_NDM–__1_*-*ΔIS*Aba125*) in a *P. mirabilis* isolate containing also a SGI1-Z genomic island (Qin et al., 2015). With the description of this strain, we might hypothesize that the chromosomal location of *bla*_NDM–__1_ gene inside a SGI or a PGI-like structure might result from consecutive genetic rearrangements starting by acquisition of a *bla*_NDM–__1_ carrying plasmid in a SGI/PGI carrying *P. mirabilis* isolate and subsequent mobilization of the Tn*125* transposon from the plasmid into the SGI/PGI.

#### Carbapenem-Hydrolyzing Class D β-Lactamases (CHDLs)

Among the Ambler class D β-lactamases only few variants with carbapenem-hydrolyzing activities have been characterized. Among them, OXA-48 is the most frequently identified in Enterobacterales. Whereas OXA-23, OXA-40, OXA-58, OXA-143 are mostly identified in *Acinetobacter baumannii* isolates.

##### OXA-48

OXA-48, is a CHDL, originally identified in a *K. pneumoniae* isolate in Turkey (Poirel et al., 2004). Acquisition of the IncL conjugative plasmid carrying the *bla*_OXA–__48_ gene has been shown to be intra and inter-species (Fursova et al., 2015). This 62.5-kb plasmid has now widely diffused among Enterobacterales (Poirel et al., 2012). Surprisingly, *Proteus* spp. OXA-48 producers have only been very rarely reported. The only descriptions of OXA-48 producing *P. mirabilis* isolates is from Palestine (strain isolated in 2012) (Chen et al., 2015) and from Russia (study reporting data from 2013 to 2014) (Fursova et al., 2015). In Moscow’s hospital, OXA-48-like carbapenemases were detected in 23.3% of *P. mirabilis* isolates due to the dissemination of one clone, and in 55.3% of *K. pneumoniae* isolates, due to the dissemination of one plasmid (Fursova et al., 2015). The Palestinian study focused on a single strain isolated from a urine sample and shows the simultaneous presence of *bla*_OXA–__48_, *bla*_CTX–M–__14_, *bla*_TEM–__1_ genes and of 13 other resistance genes including resistance to aminoglycosides [*aph(3)-Ia, aadA1, aac(3)-IIa, aph(3)-VIb, strA, strB*], fluoroquinolones (*qnrD*), streptothricin (*sat-1*), phenicols (*catA1 and cat*), tetracycline *tet*(*J*), sulfonamide-trimethoprim (*sul2* and *dfrA1*), and 27 genes related to the resistance to antiseptics and toxic products, including *arsR* (arsenic), *cutEF* (copper), *merA* (mercury), and *emrD* (benzalkonium chloride) (Chen et al., 2015) (Figure 3).

Besides the OXA-48 family, which is exclusively found in Enterobacterales and is thus expected to be found in *Proteus*, other CHDLs such as OXA-23, OXA-40, OXA-58 that have been exclusively identified in *Acinetobacter* species (Bonnin et al., 2013a) have recently been identified in *P. mirabilis* (Bonnet et al., 2002; Girlich et al., 2017; Leulmi et al., 2019; Potron et al., 2019).

##### OXA-23

OXA-23 is the most widespread CHDL in the world. The progenitor of this gene has been identified in *Acinetobacter radioresistens* (Poirel et al., 2008). The first reported OXA-23-producing (formerly ARI-1) isolate was a carbapenem-resistant *A. baumannii* detected in Scotland in 1985 (Paton et al., 1993). In this first study, the *bla*_OXA–__23_ gene in *A. baumannii* was plasmid located (Paton et al., 1993). In *A. baumannii* the *bla*_OXA–__23_ gene can be located on different transposons Tn*2006*, Tn*2007* and Tn*2008* (Mugnier et al., 2010). OXA-23 has since been widely detected, always in *Acinetobacter* isolates, until 2002, when Bonnet et al. (2002) detected this carbapenemase in *P. mirabilis*. This study investigated the persistence over 3 years of an OXA-23-producing *P. mirabilis*, in different care units in France (1996 to 1999) (Bonnet et al., 2002). In *Proteus* spp. OXA-23 confers resistance to amoxicillin, amoxicillin + clavulanate, ticarcillin, ticarcilline + clavulanate, and a decreased susceptibility to cephalotin, cefpirome and carbapenems (Table 1). In study of Bonnet et al. (2002) the *bla*_OXA–__23_ gene was chromosome-encoded, probably resulting from the integration of a plasmid unable to replicate in Enterobacterales. More recently, Österblad et al. (2016) in Finland, and Potron et al. (2019) in France also described the occurrence of the *bla*_OXA–__23_ gene in the chromosome of a *P. mirabilis* isolate with a close genetic environment typical of *A. baumannii* with insertion sequences IS*Aba1*, IS*Aba14* and IS*Aba125*. Other resistance genes would have been inserted together with *bla*_OXA–__23_ such as *sul2, floR, strB*, and *strA* (Österblad et al., 2016). In addition, this *bla*_OXA–__23_ gene, thought to be restricted to the *A. baumannii* species, was also incidentally identified on an *E. coli* plasmid in Singapore (La et al., 2014). Considering the presence of transfer elements and the relatively low hydrolytic activity toward carbapenems conferred by this enzyme, it is feared that the *bla*_OXA–__23_ genes have already silently disseminated among Enterobacterales, especially in *Proteus* spp. Indeed, our recent data (RB, DG, LD, TN) suggest that OXA-23 is increasingly identified in *Proteus* spp., although it is mostly ignored because of the low level of resistance conferred by this enzyme.

##### OXA-58

OXA-58 is a widespread CHDL in imipenem-resistant *Acinetobacter* spp., but has also been described outside the genus *Acinetobacter*, in *K. pneumoniae, E. coli, Pseudomonas* spp. and *Burkholderia* spp. in Sierra Leone (Moland et al., 2006). The *bla*_OXA–__58_ gene may be chromosome- or plasmid-encoded (Bonnin et al., 2013a). We have recently described in *P. mirabilis* the chromosomal location with tandem repeats of a fragment containing simultaneously *bla*_OXA–__58_, *bla*_AmpC_ genes of an uncultured species and a truncated IS*Aba3* (Girlich et al., 2017). The integration of these fragments took place at the XerC-XerD recombinase recognition site (Girlich et al., 2017). Tandem amplifications often involve repeat elements, insertion sequences, or rRNA (Brochet et al., 2008) operons (Figure 3 and Table 1). In this *P. mirabilis* isolate OXA-58 conferred resistance to temocillin, as well as resistance to the piperacillin-tazobactam combination and a slight decrease in susceptibility to ertapenem (MIC 0.125 mg/L) (Girlich et al., 2017). The association of these three features is a good marker of the presence of and OXA-like carbapenemase.

##### OXA-24/40

The *bla*_OXA–__24__/__40_ gene was identified initially in 1997 from isolates that were part of an outbreak in Spain, and since then it has never been detected in strains other than *A. baumannii* and mostly on the Iberic peninsula (Evans and Amyes, 2014). However, very recently, an MDR *P. mirabilis* isolate harboring the *bla*_OXA–__24__/__40_ gene, *armA* 16S rRNA methylase and *aac*(6)*-Ib-cr* genes was found for the first time in Algeria (Moland et al., 2006).

### Mechanisms of Non-enzymatic β-Lactam Resistance

The relative non-susceptibility of *Morganellaceae* to imipenem has not caused major clinical problems, but the inability of imipenem to eradicate *P. mirabilis* from soft-tissue infections was reported, as did post-treatment colonization by the organism (Dirksen et al., 1986). Some *P. mirabilis* isolates are more resistant, with imipenem MICs ranging from 16 to 64 mg/L, and one step mutants with these resistance levels can be isolated *in vitro*. However, Villar (1997) showed that loss of the 26 kDa OMP and decreased expression of the 41 and 44 kDa OMP did not themselves confer resistance to these compounds. Rather, some evidence suggest that the imipenem resistance reflects changes in penicillin binding proteins (PBP2) (Villar, 1997). PBP 2 production was expected to decline, which may explain the cross-resistance to imipenem and mecillinam. Neuwirth et al. showed that this was not the case and instead observed a decreased affinity of PBP2 for imipenem and a smaller amount of PBP1A (Neuwirth et al., 1995). Tsai et al. (2015) identified that the ImpR OMP, increased in *P. mirabilis hfq* mutant, contributed to a decrease in carbapenem susceptibility. ImpR was a homolog of *Salmonella* YbfM, a porin for chitobiose and subjected to MicM (a small RNA) regulation (Tsai et al., 2015). Raimondi et al. (1991) showed that deficiency in a major outer membrane protein of 37 kDa, was responsible for the very low permeability of the membrane of *P. rettgeri* and thus, for high-level resistance to both meropenem and imipenem. This protein was earlier identified as the major non-specific porin of 40 kDa in *P. mirabilis* and of 37 kDa in *P. vulgaris, M. morganii, P. rettgeri*, and *Providencia alcalifaciens* (Mitsuyama et al., 1987). The lack of this porin resulted in a marked decrease in susceptibility to tetracycline and cephalosporins, except cefoperazone (Mitsuyama et al., 1987).

## Potential *P. mirabilis* Animal-To-Human Transmission

The spread of SGI1/PGI1 in multidrug resistant *P. mirabilis* isolates in animals does not seem to be a sporadic phenomenon and should be the subject of sustained attention. According to Schultz et al. (2015), the sudden high prevalence of SGI/PGI1-containing *Proteus* spp. in veterinary studies suggests (i) either that these elements have not been detected in previous studies on *Proteus* spp. or (ii) a very rapid dissemination of these strains since their first description in 2011 (Siebor and Neuwirth, 2011). These data also suggest the possible way of transmission between man and pet or man and animals from food. *P. mirabilis* should be considered by hygiene committees as a potential reservoir of resistance genes in pets. In addition to the risk of animal-to-human transmission, there is a risk of gene transmission between bacterial species *via* SGI/PGI and plasmids as this was the case between *P. mirabilis* and other Enterobacterales or other non-related species such as *A. baumannii*.

## Conclusion

*Proteus mirabilis* can be found in a wide variety of environments, including soil, water sources, and sewage, but it is predominantly a commensal of the gastrointestinal tracts of humans and animals. *P. mirabilis* is a bacterium of medical importance and usually responsible for most of the common nosocomial opportunistic infections, including those of wounds, the eye, the gastrointestinal tract, and the urinary tract, it is an agent of catheter biofilm formation, quickly fouling the surface of a newly inserted urinary catheter. This review aimed to collect all the genetic mechanisms associated with the multidrug resistance phenotype of *Proteus* spp. focusing on *P. mirabilis.* A second objective was to highlight the potential reservoir role of these species for antibiotic resistance genes. We have reported globally the emergence and prevalence of these determinants of antimicrobial resistance in certain bacteria and their acquisition by *Proteus* spp. Although Armbruster et al. (2018) postulated that plasmids are not a defining feature for *P. mirabilis*, we have listed here several plasmids harboring antibiotic resistance genes including resistance to aminoglycosides, to quinolones and to β-lactams within conjugative and non-conjugative but mobilizable elements (*mic*). Concerning antibiotic resistance determinants, similarly to *Salmonella* spp., *P. mirabilis* carries integrative and conjugative elements (ICEPm) and other ICEs in the SXT/R391 family that can self-replicate and self-transfer to other strains and species, transferring virulence genes and antibiotic resistance. One of the best examples are genetic exchanges that occurred between an *Acinetobacter* species and *P. mirabilis*, resulting in the acquisition and expression of two β-lactamase genes, *bla*_OXA–__58_ and *bla*_AmpC_, at the same chromosomal locus.

## Author Contributions

All authors listed have made a substantial, direct and intellectual contribution to the work, and approved it for publication.

## Conflict of Interest

The authors declare that the research was conducted in the absence of any commercial or financial relationships that could be construed as a potential conflict of interest.

## Footnotes

1 http://www.bldb.eu/

## References

[B1] Aberkane S. Compain F. Decré D. Dupont C. Laurens C. Vittecoq M. (2016). High prevalence of SXT/R391-related integrative and conjugative elements carrying *bla*CMY-2 in *Proteus mirabilis* isolates from gulls in the south of France. *Antimicrob. Agents Chemother.* 60 1148–1152. 10.1128/AAC.01654-15 26643344 PMC4750665

[B2] Abreu A. G. Marques S. G. Monteiro-Neto V. de Carvalho R. M. L. Gonçalves A. G. (2011). Nosocomial infection and characterization of extended-spectrum β-lactamases-producing *Enterobacteriaceae* in Northeast Brazil. *Rev. Soc. Bras. Med. Trop.* 44 441–446. 10.1590/s0037-86822011000400008 21860889

[B3] Adeolu M. Alnajar S. Naushad S. S Gupta R. (2016). Genome-based phylogeny and taxonomy of the ‘*Enterobacteriales*’: proposal for *Enterobacterales* ord. nov. divided into the families *Enterobacteriaceae*, *Erwiniaceae* fam. nov., *Pectobacteriaceae* fam. nov., *Yersiniaceae* fam. nov., *Hafniaceae* fam. nov., *Morganellaceae* fam. nov., and *Budviciaceae* fam. nov. *Int. J. Syst. Evol. Microbiol.* 66 5575–5599. 10.1099/ijsem.0.001485 27620848

[B4] Ahmed A. M. Hussein A. I. A. Shimamoto T. (2007). *Proteus mirabilis* clinical isolate harbouring a new variant of *Salmonella* genomic island 1 containing the multiple antibiotic resistance region. *J. Antimicrob. Chemother.* 59 184–190. 10.1093/jac/dkl471 17114173

[B5] Alabi O. S. Mendonça N. Adeleke O. E. Da Silva G. J. (2017). Molecular screening of antibiotic-resistant determinants among multidrug-resistant clinical isolates of *Proteus mirabilis* from SouthWest Nigeria. *Afr. Health Sci.* 17 356–365. 10.4314/ahs.v17i2.9 29062330 PMC5637020

[B6] Albornoz E. Lucero C. Romero G. Rapoport M. Guerriero L. Andres P. (2014). Analysis of plasmid-mediated quinolone resistance genes in clinical isolates of the tribe *Proteeae* from Argentina: first report of *qnrD* in the Americas. *J. Glob. Antimicrob. Resist.* 2 322–326. 10.1016/j.jgar.2014.05.005 27873695

[B7] Ambler R. P. (1980). The structure of beta-lactamases. *Philos. Trans. R. Soc. Lond. B Biol. Sci.* 289 321–331. 6109327 10.1098/rstb.1980.0049

[B8] Armbruster C. E. Mobley H. L. T. Pearson M. M. (2018). Pathogenesis of *Proteus mirabilis* infection. *EcoSal Plus* 8: 10.1128/ecosalplus.ESP-0009-2017. PMC5880328 29424333

[B9] Arpin C. Dubois V. Coulange L. André C. Fischer I. Noury P. (2003). Extended-spectrum β-lactamase-producing *Enterobacteriaceae* in community and private health care centers. *Antimicrob. Agents Chemother.* 47 3506–3514. 10.1128/aac.47.11.3506-3514.2003 14576109 PMC253776

[B10] Bahar G. Eraç B. Mert A. Gülay Z. (2004). PER-1 production in a urinary isolate of *Providencia rettgeri*. *J. Chemother.* 16 343–346. 10.1179/joc.2004.16.4.343 15332708

[B11] Bailey J. K. Pinyon J. L. Anantham S. Hall R. M. (2011). Distribution of the *bla*TEM gene and *bla*TEM-containing transposons in commensal *Escherichia coli*. *J. Antimicrob. Chemother.* 66 745–751. 10.1093/jac/dkq529 21393132

[B12] Baraniak A. Izdebski R. Fiett J. Gawryszewska I. Bojarska K. Herda M. (2016). NDM-producing *Enterobacteriaceae* in Poland, 2012-14: inter-regional outbreak of *Klebsiella pneumoniae* ST11 and sporadic cases. *J. Antimicrob. Chemother.* 71 85–91. 10.1093/jac/dkv282 26386745

[B13] Bauernfeind A. Schweighart S. Chong Y. (1989). Extended broad spectrum β-lactamase in *Klebsiella pneumoniae* including resistance to cephamycins. *Infection* 17 316–321. 10.1007/bf01650718 2689349

[B14] Bauernfeind A. Stemplinger I. Jungwirth R. Ernst S. Casellas J. M. (1996a). Sequences of beta-lactamase genes encoding CTX-M-1 (MEN-1) and CTX-M-2 and relationship of their amino acid sequences with those of other beta-lactamases. *Antimicrob. Agents Chemother.* 40 509–513. 10.1128/aac.40.2.509 8834913 PMC163149

[B15] Bauernfeind A. Stemplinger I. Jungwirth R. Mangold P. Amann S. Akalin E. (1996b). Characterization of beta-lactamase gene *bla*PER-2, which encodes an extended-spectrum class A beta-lactamase. *Antimicrob. Agents Chemother.* 40 616–620. 10.1128/aac.40.3.616 8851581 PMC163168

[B16] Bauernfeind A. Wagner S. Jungwirth R. Schneider I. Meyer D. (1997). A novel class C beta-lactamase FOX-2 in *Escherichia coli* conferring resistance to cephamycins. *Antimicrob. Agents Chemother.* 41 2041–2046. 10.1128/aac.41.9.2041 9303413 PMC164064

[B17] Bidet P. Verdet C. Gautier V. Bingen E. Arlet G. (2005). First description of DHA-1 AmpC beta-lactamase in *Proteus mirabilis*. *Clin. Microbiol. Infect.* 11 591–592. 10.1111/j.1469-0691.2005.01178.x 15966982

[B18] Biedenbach D. J. Bouchillon S. K. Hoban D. J. Hackel M. Phuong D. M. Nga T. T. T. (2014). Antimicrobial susceptibility and extended-spectrum beta-lactamase rates in aerobic gram-negative bacteria causing intra-abdominal infections in Vietnam: report from the Study for Monitoring Antimicrobial Resistance Trends (SMART 2009–2011). *Diagn. Microbiol. Infect. Dis.* 79 463–467. 10.1016/j.diagmicrobio.2014.05.009 24923210

[B19] Biendo M. Thomas D. Laurans G. Hamdad-Daoudi F. Canarelli B. Rousseau F. (2005). Molecular diversity of *Proteus mirabilis* isolates producing extended-spectrum beta-lactamases in a French university hospital. *Clin. Microbiol. Infect.* 11 395–401. 10.1111/j.1469-0691.2005.01147.x 15819867

[B20] Bonnet R. (2004). Growing group of extended-spectrum beta-lactamases: the CTX-M enzymes. *Antimicrob. Agents Chemother.* 48 1–14. 10.1128/aac.48.1.1-14.2004 14693512 PMC310187

[B21] Bonnet R. Marchandin H. Chanal C. Sirot D. Labia R. De Champs C. (2002). Chromosome-encoded class D beta-lactamase OXA-23 in *Proteus mirabilis*. *Antimicrob. Agents Chemother.* 46 2004–2006. 10.1128/aac.46.6.2004-2006.2002 12019126 PMC127228

[B22] Bonnet R. Sampaio J. L. Labia R. De Champs C. Sirot D. Chanal C. (2000). A novel CTX-M beta-lactamase (CTX-M-8) in cefotaxime-resistant *Enterobacteriaceae* isolated in Brazil. *Antimicrob. Agents Chemother.* 44 1936–1942. 10.1128/aac.44.7.1936-1942.2000 10858358 PMC89989

[B23] Bonnin R. A. Nordmann P. Poirel L. (2013a). Screening and deciphering antibiotic resistance in *Acinetobacter baumannii*: a state of the art. *Expert Rev. Anti Infect. Ther.* 11 571–583. 10.1586/eri.13.38 23750729

[B24] Bonnin R. A. Poirel L. Benoit-Cattin T. Nordmann P. (2013b). Ceftazidime-susceptible and imipenem-non-susceptible OXA-58-producing *Acinetobacter baumannii* from the Comoros archipelago. *Int. J. Antimicrob. Agents* 41 297–298. 10.1016/j.ijantimicag.2012.11.002 23313400

[B25] Bret L. Chanal C. Sirot D. Labia R. Sirot J. (1996). Characterization of an inhibitor-resistant enzyme IRT-2 derived from TEM-2 beta-lactamase produced by *Proteus mirabilis* strains. *J. Antimicrob. Chemother.* 38 183–191. 10.1093/jac/38.2.183 8877532

[B26] Bret L. Chanal-Claris C. Sirot D. Chaibi E. B. Labia R. Sirot J. (1998). Chromosomally encoded AmpC-type β-lactamase in a clinical isolate of *Proteus mirabilis*. *Antimicrob. Agents Chemother.* 42 1110–1114. 10.1128/aac.42.5.1110 9593136 PMC105754

[B27] Brochet M. Couvé E. Zouine M. Poyart C. Glaser P. (2008). A naturally occurring gene amplification leading to sulfonamide and trimethoprim resistance in *Streptococcus agalactiae*. *J. Bacteriol.* 190 672–680. 10.1128/jb.01357-07 18024520 PMC2223700

[B28] Bush K. Jacoby G. A. (2010). Updated functional classification of beta-lactamases. *Antimicrob. Agents Chemother.* 54 969–976. 10.1128/AAC.01009-09 19995920 PMC2825993

[B29] Cabral A. B. Maciel M. A. V. Barros J. F. Antunes M. M. Lopes A. C. S. (2015). Detection of *bla*KPC-2 in *Proteus mirabilis* in Brazil. *Rev. Soc. Bras. Med. Trop.* 48 94–95. 10.1016/j.jgar.2019.08.026 25860472

[B30] Cambau E. Lascols C. Sougakoff W. Bébéar C. Bonnet R. Cavallo J.-D. (2006). Occurrence of *qnrA*-positive clinical isolates in French teaching hospitals during 2002-2005. *Clin. Microbiol. Infect.* 12 1013–1020. 10.1111/j.1469-0691.2006.01529.x 16961639

[B31] Cantón R. Loza E. Aznar J. Castillo F. J. Cercenado E. Fraile-Ribot P. A. (2019). Monitoring the antimicrobial susceptibility of Gram-negative organisms involved in intraabdominal and urinary tract infections recovered during the SMART study (Spain, 2016 and 2017). *Rev. Esp. Quimioter.* 32 145–155. 30761824 PMC6441989

[B32] Carattoli A. (2009). Resistance plasmid families in *Enterobacteriaceae*. *Antimicrob. Agents Chemother.* 53 2227–2238. 10.1128/aac.01707-08 19307361 PMC2687249

[B33] Castanheira M. Mills J. C. Costello S. E. Jones R. N. Sader H. S. (2015). Ceftazidime-avibactam activity tested against *Enterobacteriaceae* isolates from U.S. hospitals (2011 to 2013) and characterization of β-lactamase-producing strains. *Antimicrob. Agents Chemother.* 59 3509–3517. 10.1128/AAC.00163-15 25845862 PMC4432207

[B34] Chanal C. Bonnet R. De Champs C. Sirot D. Labia R. Sirot J. (2000). Prevalence of beta-lactamases among 1,072 clinical strains of *Proteus mirabilis*: a 2-year survey in a French hospital. *Antimicrob. Agents Chemother.* 44 1930–1935. 10.1128/aac.44.7.1930-1935.2000 10858357 PMC89988

[B35] Chen L. Laham N. A. Chavda K. D. Mediavilla J. R. Jacobs M. R. Bonomo R. A. (2015). First report of an OXA-48-producing multidrug-resistant *Proteus mirabilis* strain from Gaza, Palestine. *Antimicrob. Agents Chemother.* 59 4305–4307. 10.1128/AAC.00565-15 25896692 PMC4468693

[B36] Choury D. Aubert G. Szajnert M. F. Azibi K. Delpech M. Paul G. (1999). Characterization and nucleotide sequence of CARB-6, a new carbenicillin-hydrolyzing beta-lactamase from *Vibrio cholerae*. *Antimicrob. Agents Chemother.* 43 297–301. 10.1128/aac.43.2.297 9925522 PMC89067

[B37] Choury D. Szajnert M.-F. Joly-Guillou M.-L. Azibi K. Delpech M. Paul G. (2000). Nucleotide sequence of the *bla*RTG-2 (CARB-5) gene and phylogeny of a new group of carbenicillinases. *Antimicrob. Agents Chemother.* 44 1070–1074. 10.1128/aac.44.4.1070-1074.2000 10722515 PMC89816

[B38] Cicek A. C. Duzgun A. O. Saral A. Sandalli C. (2014). Determination of a novel integron-located variant (*bla*OXA -320) of class D β-lactamase in *Proteus mirabilis*: a novel integron-located *bla*OXA- 320 in *Proteus mirabilis*. *J. Basic Microbiol.* 54 1030–1035. 10.1002/jobm.201300264 24027220

[B39] Cuzon G. Naas T. Villegas M.-V. Correa A. Quinn J. P. Nordmann P. (2011). Wide dissemination of *Pseudomonas aeruginosa* producing beta-lactamase *bla*KPC-2 gene in Colombia. *Antimicrob. Agents Chemother.* 55 5350–5353. 10.1128/AAC.00297-11 21844315 PMC3195029

[B40] Dahmen S. Madec J.-Y. Haenni M. (2013). F2:A-:B- plasmid carrying the extended-spectrum β-lactamase *bla*CTX-M-55/57 gene in *Proteus mirabilis* isolated from a primate. *Int. J. Antimicrob. Agents* 41 594–595. 10.1016/j.ijantimicag.2013.02.004 23507413

[B41] D’Andrea M. M. Literacka E. Zioga A. Giani T. Baraniak A. Fiett J. (2011). Evolution and spread of a multidrug-resistant *Proteus mirabilis* clone with chromosomal AmpC-type cephalosporinases in Europe. *Antimicrob. Agents Chemother.* 55 2735–2742. 10.1128/AAC.01736-10 21402851 PMC3101460

[B42] D’Andrea M. M. Nucleo E. Luzzaro F. Giani T. Migliavacca R. Vailati F. (2006). CMY-16, a novel acquired AmpC-type beta-lactamase of the CMY/LAT lineage in multifocal monophyletic isolates of *Proteus mirabilis* from northern Italy. *Antimicrob. Agents Chemother.* 50 618–624. 10.1128/aac.50.2.618-624.2006 16436718 PMC1366893

[B43] Datta P. Gupta V. Arora S. Garg S. Chander J. (2014). Epidemiology of extended-spectrum β-Lactamase, AmpC, and carbapenemase production in *Proteus mirabilis*. *Jpn. J. Infect. Dis.* 67 44–46. 10.7883/yoken.67.44 24451101

[B44] de Champs C. Bonnet R. Sirot D. Chanal C. Sirot J. (2000). Clinical relevance of *Proteus mirabilis* in hospital patients: a two year survey. *J. Antimicrob. Chemother.* 45 537–539. 10.1093/jac/45.4.537 10747835

[B45] Decré D. Verdet C. Raskine L. Blanchard H. Burghoffer B. Philippon A. (2002). Characterization of CMY-type beta-lactamases in clinical strains of *Proteus mirabilis* and *Klebsiella pneumoniae* isolated in four hospitals in the Paris area. *J. Antimicrob. Chemother.* 50 681–688. 10.1093/jac/dkf193 12407124

[B46] Di Pilato V. Chiarelli A. Boinett C. J. Riccobono E. Harris S. R. D’Andrea M. M. (2016). Complete genome sequence of the first KPC-type carbapenemase-positive *Proteus mirabilis* strain from a bloodstream infection. *Genome Announc.* 4:e00607-16. 10.1128/genomeA.00607-16 27340072 PMC4919411

[B47] Dirksen M. S. Wintermans R. G. Boerema J. B. Gimbrère J. S. (1986). Imipenem as monotherapy in the treatment of intensive care patients with severe infections. *J. Antimicrob. Chemother.* 18(Suppl. E), 145–151. 10.1093/jac/18.supplement_e.145 3469187

[B48] Dixon N. Fowler R. C. Yoshizumi A. Horiyama T. Ishii Y. Harrison L. (2016). IMP-27, a unique metallo-β-lactamase identified in geographically distinct isolates of *Proteus mirabilis*. *Antimicrob. Agents Chemother.* 60 6418–6421. 10.1128/AAC.02945-15 27503648 PMC5038328

[B49] Dortet L. Girlich D. Virlouvet A.-L. Poirel L. Nordmann P. Iorga B. I. (2017). Characterization of BRPMBL, the bleomycin-resistance protein associated with the carbapenemase NDM. *Antimicrob. Agents Chemother.* 61:e02413-16. 10.1128/AAC.02413-16 28069656 PMC5328552

[B50] Dortet L. Poirel L. Al Yaqoubi F. Nordmann P. (2012). NDM-1, OXA-48 and OXA-181 carbapenemase-producing *Enterobacteriaceae* in Sultanate of Oman. *Clin. Microbiol. Infect.* 18 E144–E148. 22404169 10.1111/j.1469-0691.2012.03796.x

[B51] Doublet B. Boyd D. Mulvey M. R. Cloeckaert A. (2005). The *Salmonella* genomic island 1 is an integrative mobilizable element. *Mol. Microbiol.* 55 1911–1924. 10.1111/j.1365-2958.2005.04520.x 15752209

[B52] Evans B. A. Amyes S. G. B. (2014). OXA -lactamases. *Clin. Microbiol. Rev.* 27 241–263. 10.1128/CMR.00117-13 24696435 PMC3993105

[B53] Fursova N. K. Astashkin E. I. Knyazeva A. I. Kartsev N. N. Leonova E. S. Ershova O. N. (2015). The spread of *bla*OXA-48 and *bla*OXA-244 carbapenemase genes among *Klebsiella pneumoniae*, *Proteus mirabilis* and *Enterobacter* spp. isolated in Moscow, Russia. *Ann. Clin. Microbiol. Antimicrob.* 14:46. 10.1186/s12941-015-0108-y PMC4630924 26526183

[B54] Gaillot O. Clément C. Simonet M. Philippon A. (1997). Novel transferable beta-lactam resistance with cephalosporinase characteristics in *Salmonella enteritidis*. *J. Antimicrob. Chemother.* 39 85–87. 10.1093/jac/39.1.85 9044032

[B55] Galani I. Souli M. Panagea T. Poulakou G. Kanellakopoulou K. Giamarellou H. (2012). Prevalence of 16S rRNA methylase genes in *Enterobacteriaceae* isolates from a Greek university hospital. *Clin. Microbiol. Infect.* 18 E52–E54. 22264302 10.1111/j.1469-0691.2011.03738.x

[B56] Giammanco G. M. Grimont P. A. D. Grimont F. Lefevre M. Giammanco G. Pignato S. (2011). Phylogenetic analysis of the genera *Proteus*, *Morganella* and *Providencia* by comparison of *rpoB* gene sequences of type and clinical strains suggests the reclassification of *Proteus myxofaciens* in a new genus, *Cosenzaea* gen. nov., as *Cosenzaea myxofaciens* comb. nov. *Int. J. Syst. Evol. Microbiol.* 61 1638–1644. 10.1099/ijs.0.021964-0 20709916

[B57] Girlich D. Bonnin R. A. Bogaerts P. De Laveleye M. Huang D. T. Dortet L. (2017). Chromosomal amplification of the *bla*OXA-58 carbapenemase gene in a *Proteus mirabilis* clinical isolate. *Antimicrob. Agents Chemother.* 61:e01697-16. 10.1128/AAC.01697-16 PMC5278702 27855079

[B58] Girlich D. Dortet L. Poirel L. Nordmann P. (2015). Integration of the *bla*NDM-1 carbapenemase gene into *Proteus* genomic island 1 (PGI1-PmPEL) in a *Proteus mirabilis* clinical isolate. *J. Antimicrob. Chemother.* 70 98–102. 10.1093/jac/dku371 25239462

[B59] Girlich D. Karim A. Spicq C. Nordmann P. (2000a). Plasmid-mediated cephalosporinase ACC-1 in clinical isolates of *Proteus mirabilis* and *Escherichia coli*. *Eur. J. Clin. Microbiol. Infect. Dis.* 19 893–895. 10.1007/s100960000386 11152321

[B60] Girlich D. Naas T. Bellais S. Poirel L. Karim A. Nordmann P. (2000b). Biochemical-genetic characterization and regulation of expression of an ACC-1-like chromosome-borne cephalosporinase from *Hafnia alvei*. *Antimicrob. Agents Chemother.* 44 1470–1478. 10.1128/aac.44.6.1470-1478.2000 10817695 PMC89899

[B61] Gonzalez Leiza M. Perez-Diaz J. C. Ayala J. Casellas J. M. Martinez-Beltran J. Bush K. (1994). Gene sequence and biochemical characterization of FOX-1 from *Klebsiella pneumoniae*, a new AmpC-type plasmid-mediated beta-lactamase with two molecular variants. *Antimicrob. Agents Chemother.* 38 2150–2157. 10.1128/aac.38.9.2150 7811034 PMC284699

[B62] Guillard T. Grillon A. de Champs C. Cartier C. Madoux J. Berçot B. (2014). Mobile insertion cassette elements found in small non-transmissible plasmids in *Proteeae* may explain *qnrD* mobilization. *PLoS One* 9:e87801. 10.1371/journal.pone.0087801 24504382 PMC3913671

[B63] Hall R. M. Collis C. M. (1995). Mobile gene cassettes and integrons: capture and spread of genes by site-specific recombination. *Mol. Microbiol.* 15 593–600. 10.1111/j.1365-2958.1995.tb02368.x 7783631

[B64] Harada S. Ishii Y. Saga T. Kouyama Y. Tateda K. Yamaguchi K. (2012). Chromosomal integration and location on IncT plasmids of the blaCTX-M-2 gene in *Proteus mirabilis* clinical isolates. *Antimicrob. Agents Chemother.* 56 1093–1096. 10.1128/AAC.00258-11 22106217 PMC3264238

[B65] Harada S. Ishii Y. Saga T. Tateda K. Yamaguchi K. (2010). Chromosomally encoded *bla*CMY-2 located on a novel SXT/R391-related integrating conjugative element in a *Proteus mirabilis* clinical isolate. *Antimicrob. Agents Chemother.* 54 3545–3550. 10.1128/AAC.00111-10 20566768 PMC2934980

[B66] Helmy M. M. Wasfi R. (2014). Phenotypic and molecular characterization of plasmid mediated AmpC β-lactamases among *Escherichia coli*, *Klebsiella* spp., and *Proteus mirabilis* isolated from urinary tract infections in Egyptian hospitals. *Biomed Res. Int.* 2014:171548. 10.1155/2014/171548 25003107 PMC4070535

[B67] Ho P. L. Ho A. Y. M. Chow K. H. Wong R. C. W. Duan R. S. Ho W. L. (2005). Occurrence and molecular analysis of extended-spectrum β-lactamase-producing *Proteus mirabilis* in Hong Kong, 1999-2002. *J. Antimicrob. Chemother.* 55 840–845. 10.1093/jac/dki135 15857942

[B68] Hu Y. Cai J. Zhang R. Zhou H. Sun Q. Chen G. (2012). Emergence of *Proteus mirabilis* harboring *bla*KPC-2 and *qnrD* in a Chinese hospital. *Antimicrob. Agents Chemother.* 56 2278–2282. 10.1128/AAC.05519-11 22354308 PMC3346625

[B69] Huang C.-W. Chien J.-H. Peng R.-Y. Tsai D.-J. Li M.-H. Lee H.-M. (2015). Molecular epidemiology of CTX-M-type extended-spectrum β-lactamase-producing *Proteus mirabilis* isolates in Taiwan. *Int. J. Antimicrob. Agents* 45 84–85. 10.1016/j.ijantimicag.2014.09.004 25446905

[B70] Jacoby G. A. (2009). AmpC beta-lactamases. *Clin. Microbiol. Rev.* 22 161–182, Tableof Contents. 10.1128/CMR.00036-08 19136439 PMC2620637

[B71] Jayol A. Janvier F. Guillard T. Chau F. Mérens A. Robert J. (2016). qnrA6 genetic environment and quinolone resistance conferred on *Proteus mirabilis*. *J. Antimicrob. Chemother.* 71 903–908. 10.1093/jac/dkv431 26747095

[B72] Jones C. H. Tuckman M. Keeney D. Ruzin A. Bradford P. A. (2009). Characterization and sequence analysis of extended-spectrum-β-lactamase-encoding genes from *Escherichia coli*, *Klebsiella pneumoniae*, and *Proteus mirabilis* isolates collected during tigecycline phase 3 clinical trials. *Antimicrob. Agents Chemother.* 53 465–475. 10.1128/AAC.00883-08 19015360 PMC2630642

[B73] Karim A. Poirel L. Nagarajan S. Nordmann P. (2001). Plasmid-mediated extended-spectrum beta-lactamase (CTX-M-3 like) from India and gene association with insertion sequence IS*Ecp1*. *FEMS Microbiol. Lett.* 201 237–241. 10.1016/s0378-1097(01)00276-2 11470367

[B74] Kim J.-Y. Park Y.-J. Kim S.-I. Kang M. W. Lee S.-O. Lee K.-Y. (2004). Nosocomial outbreak by *Proteus mirabilis* producing extended-spectrum beta-lactamase VEB-1 in a Korean university hospital. *J. Antimicrob. Chemother.* 54 1144–1147. 10.1093/jac/dkh486 15546971

[B75] Knothe H. Shah P. Krcmery V. Antal M. Mitsuhashi S. (1983). Transferable resistance to cefotaxime, cefoxitin, cefamandole and cefuroxime in clinical isolates of *Klebsiella pneumoniae* and *Serratia marcescens*. *Infection* 11 315–317. 10.1007/bf01641355 6321357

[B76] La M.-V. Jureen R. Lin R. T. P. Teo J. W. P. (2014). Unusual detection of an *Acinetobacter* class D carbapenemase gene, *bla*OXA-23, in a clinical *Escherichia coli* isolate. *J. Clin. Microbiol.* 52 3822–3823. 25031438 10.1128/JCM.01566-14 PMC4187746

[B77] Labia R. Guionie M. Barthélémy M. (1981). Properties of three carbenicillin-hydrolysing beta-lactamases (CARB) from *Pseudomonas aeruginosa*: identification of a new enzyme. *J. Antimicrob. Chemother.* 7 49–56. 10.1093/jac/7.1.49 6782068

[B78] Lartigue M.-F. Fortineau N. Nordmann P. (2005). Spread of novel expanded-spectrum beta-lactamases in *Enterobacteriaceae* in a university hospital in the Paris area, France. *Clin. Microbiol. Infect.* 11 588–591. 10.1111/j.1469-0691.2005.01172.x 15966981

[B79] Lauretti L. Riccio M. L. Mazzariol A. Cornaglia G. Amicosante G. Fontana R. (1999). Cloning and characterization of *bla*VIM, a new integron-borne metallo-beta-lactamase gene from a *Pseudomonas aeruginosa* clinical isolate. *Antimicrob. Agents Chemother.* 43 1584–1590. 10.1128/aac.43.7.1584 10390207 PMC89328

[B80] Leulmi Z. Kandouli C. Mihoubi I. Benlabed K. Lezzar A. Rolain J.-M. (2019). First report of *bla*OXA-24 carbapenemase gene, *armA* methyltransferase and *aac(6’)-Ib-cr* among multidrug-resistant clinical isolates of *Proteus mirabilis* in Algeria. *J. Glob. Antimicrob. Resist.* 16 125–129. 10.1016/j.jgar.2018.08.019 30217548

[B81] Li X. Du Y. Du P. Dai H. Fang Y. Li Z. (2016). SXT/R391 integrative and conjugative elements in *Proteus* species reveal abundant genetic diversity and multidrug resistance. *Sci. Rep.* 6:37372. 10.1038/srep37372 27892525 PMC5124997

[B82] Literacka E. Empel J. Baraniak A. Sadowy E. Hryniewicz W. Gniadkowski M. (2004). Four variants of the *Citrobacter freundii* AmpC-type cephalosporinases, including novel enzymes CMY-14 and CMY-15, in a *Proteus mirabilis* clone widespread in Poland. *Antimicrob. Agents Chemother.* 48 4136–4143. 10.1128/aac.48.11.4136-4143.2004 15504832 PMC525428

[B83] Liu Z. Li W. Wang J. Pan J. Sun S. Yu Y. (2013). Identification and characterization of the first *Escherichia coli* strain carrying NDM-1 gene in China. *PLoS One* 8:e66666. 10.1371/journal.pone.0066666 23762496 PMC3677923

[B84] Luzzaro F. Perilli M. Amicosante G. Lombardi G. Belloni R. Zollo A. (2001). Properties of multidrug-resistant, ESBL-producing *Proteus mirabilis* isolates and possible role of beta-lactam/beta-lactamase inhibitor combinations. *Int. J. Antimicrob. Agents* 17 131–135. 10.1016/s0924-8579(00)00325-3 11165117

[B85] Mahillon J. Chandler M. (1998). Insertion sequences. *Microbiol. Mol. Biol. Rev.* 62 725–774. 9729608 10.1128/mmbr.62.3.725-774.1998 PMC98933

[B86] Manoharan A. Sugumar M. Kumar A. Jose H. Mathai D. Khilnani G. C. (2012). Phenotypic & molecular characterization of AmpC β-lactamases among *Escherichia coli*, *Klebsiella* spp. & *Enterobacter* spp. from five Indian medical centers. *Indian J. Med. Res.* 135 359–364. 22561623 PMC3361873

[B87] Mariotte S. Nordmann P. Nicolas M. H. (1994). Extended-spectrum beta-lactamase in *Proteus mirabilis*. *J. Antimicrob. Chemother.* 33 925–935. 10.1093/jac/33.5.925 8089066

[B88] Markovska R. Schneider I. Keuleyan E. Ivanova D. Lesseva M. Stoeva T. (2017). Dissemination of a multidrug-resistant VIM-1- and CMY-99-producing *Proteus mirabilis* clone in Bulgaria. *Microb. Drug Resist.* 23 345–350. 10.1089/mdr.2016.0026 27341161

[B89] Mata C. Miró E. Rivera A. Mirelis B. Coll P. Navarro F. (2010). Prevalence of acquired AmpC beta-lactamases in *Enterobacteriaceae* lacking inducible chromosomal *ampC* genes at a Spanish hospital from 1999 to 2007. *Clin. Microbiol. Infect.* 16 472–476. 10.1111/j.1469-0691.2009.02864.x 19523051

[B90] Mata C. Navarro F. Miró E. Walsh T. R. Mirelis B. Toleman M. (2011). Prevalence of SXT/R391-like integrative and conjugative elements carrying *bla*CMY-2 in *Proteus mirabilis*. *J. Antimicrob. Chemother.* 66 2266–2270. 10.1093/jac/dkr286 21752830

[B91] Matsumoto Y. Ikeda F. Kamimura T. Yokota Y. Mine Y. (1988). Novel plasmid-mediated beta-lactamase from *Escherichia coli* that inactivates oxyimino-cephalosporins. *Antimicrob. Agents Chemother.* 32 1243–1246. 10.1128/aac.32.8.1243 3056257 PMC172385

[B92] Mazzariol A. Kocsis B. Koncan R. Kocsis E. Lanzafame P. Cornaglia G. (2012). Description and plasmid characterization of *qnrD* determinants in *Proteus mirabilis* and *Morganella morganii*. *Clin. Microbiol. Infect.* 18 E46–E48. 10.1111/j.1469-0691.2011.03728.x 22192340

[B93] Mendes R. E. Castanheira M. Woosley L. N. Stone G. G. Bradford P. A. Flamm R. K. (2019). Characterization of β-lactamase content of ceftazidime-resistant pathogens recovered during the pathogen-directed phase 3 REPRISE Trial for ceftazidime-avibactam: correlation of efficacy against β-lactamase producers. *Antimicrob. Agents Chemother.* 63:e02655-18. 10.1128/AAC.02655-18 30910899 PMC6535560

[B94] Mitsuyama J. Hiruma R. Yamaguchi A. Sawai T. (1987). Identification of porins in outer membrane of *Proteus*, *Morganella*, and *Providencia* spp. and their role in outer membrane permeation of beta-lactams. *Antimicrob. Agents Chemother.* 31 379–384. 10.1128/aac.31.3.379 3034144 PMC174736

[B95] Mojica M. F. Bonomo R. A. Fast W. (2016). B1-metallo-β-lactamases: where do we stand? *Curr. Drug Targets* 17 1029–1050. 10.2174/1389450116666151001105622 26424398 PMC4814356

[B96] Mokracka J. Gruszczyńska B. Kaznowski A. (2012). Integrons, β-lactamase and *qnr* genes in multidrug resistant clinical isolates of *Proteus mirabilis* and *P. vulgaris*. *APMIS* 120 950–958. 10.1111/j.1600-0463.2012.02923.x 23030307

[B97] Moland E. S. Hanson N. D. Black J. A. Hossain A. Song W. Thomson K. S. (2006). Prevalence of newer β-lactamases in Gram-negative clinical isolates collected in the United States from 2001 to 2002. *J. Clin. Microbiol.* 44 3318–3324. 10.1128/jcm.00756-06 16954267 PMC1594717

[B98] Mollenkopf D. F. Stull J. W. Mathys D. A. Bowman A. S. Feicht S. M. Grooters S. V. (2017). Carbapenemase-producing *Enterobacteriaceae* recovered from the environment of a swine farrow-to-finish operation in the United States. *Antimicrob. Agents Chemother.* 61:e01298-16. 10.1128/AAC.01298-16 27919894 PMC5278694

[B99] Mugnier P. D. Poirel L. Naas T. Nordmann P. (2010). Worldwide dissemination of the *bla*OXA-23 carbapenemase gene of *Acinetobacter baumannii*. *Emerg. Infect. Dis.* 16 35–40. 10.3201/eid1601.090852 20031040 PMC2874364

[B100] Naas T. Benaoudia F. Massuard S. Nordmann P. (2000). Integron-located VEB-1 extended-spectrum beta-lactamase gene in a *Proteus mirabilis* clinical isolate from Vietnam. *J. Antimicrob. Chemother.* 46 703–711. 10.1093/jac/46.5.703 11062188

[B101] Naas T. Cuzon G. Villegas M.-V. Lartigue M.-F. Quinn J. P. Nordmann P. (2008). Genetic structures at the origin of acquisition of the beta-lactamase *bla*KPC gene. *Antimicrob. Agents Chemother.* 52 1257–1263. 10.1128/AAC.01451-07 18227185 PMC2292522

[B102] Naas T. Oueslati S. Bonnin R. A. Dabos M. L. Zavala A. Dortet L. (2017). Beta-lactamase database (BLDB) – structure and function. *J. Enzyme Inhib. Med. Chem.* 32 917–919. 10.1080/14756366.2017.1344235 28719998 PMC6445328

[B103] Naas T. Zerbib M. Girlich D. Nordmann P. (2003). Integration of a transposon Tn1-encoded inhibitor-resistant beta-lactamase gene, *bla*TEM-67 from *Proteus mirabilis*, into the *Escherichia coli* chromosome. *Antimicrob. Agents Chemother.* 47 19–26. 10.1128/aac.47.1.19-26.2003 12499163 PMC148959

[B104] Nagano N. Shibata N. Saitou Y. Nagano Y. Arakawa Y. (2003). Nosocomial outbreak of infections by *Proteus mirabilis* that produces extended-spectrum CTX-M-2 type beta-lactamase. *J. Clin. Microbiol.* 41 5530–5536. 10.1128/jcm.41.12.5530-5536.2003 14662935 PMC308985

[B105] Nakama R. Shingaki A. Miyazato H. Higa R. Nagamoto C. Hamamoto K. (2016). Current status of extended spectrum β-lactamase-producing *Escherichia coli*, *Klebsiella pneumoniae* and *Proteus mirabilis* in Okinawa prefecture, Japan. *J. Infect. Chemother.* 22 281–286. 10.1016/j.jiac.2016.01.008 26898665

[B106] Nakano R. Nakano A. Abe M. Inoue M. Okamoto R. (2012). Regional outbreak of CTX-M-2 β-lactamase-producing *Proteus mirabilis* in Japan. *J. Med. Microbiol.* 61 1727–1735. 10.1099/jmm.0.049726-0 22935848

[B107] Neuwirth C. Siébor E. Duez J. M. Péchinot A. Kazmierczak A. (1995). Imipenem resistance in clinical isolates of *Proteus mirabilis* associated with alterations in penicillin-binding proteins. *J. Antimicrob. Chemother.* 36 335–342. 10.1093/jac/36.2.335 8522463

[B108] Nijssen S. Florijn A. Bonten M. J. M. Schmitz F. J. Verhoef J. Fluit A. C. (2004). Beta-lactam susceptibilities and prevalence of ESBL-producing isolates among more than 5000 European *Enterobacteriaceae* isolates. *Int. J. Antimicrob. Agents* 24 585–591. 10.1016/j.ijantimicag.2004.08.008 15555882

[B109] Nishio H. Komatsu M. Shibata N. Shimakawa K. Sueyoshi N. Ura T. (2004). Metallo-β-lactamase-producing Gram-negative bacilli: laboratory-based surveillance in cooperation with 13 clinical laboratories in the Kinki region of Japan. *J. Clin. Microbiol.* 42 5256–5263. 10.1128/jcm.42.11.5256-5263.2004 15528723 PMC525181

[B110] Nordmann P. Naas T. (1994). Sequence analysis of PER-1 extended-spectrum beta-lactamase from *Pseudomonas aeruginosa* and comparison with class A beta-lactamases. *Antimicrob. Agents Chemother.* 38 104–114. 10.1128/aac.38.1.104 8141562 PMC284404

[B111] Ogbolu D. O. Daini O. A. Ogunledun A. Alli A. O. Webber M. A. (2011). High levels of multidrug resistance in clinical isolates of Gram-negative pathogens from Nigeria. *Int. J. Antimicrob. Agents* 37 62–66. 10.1016/j.ijantimicag.2010.08.019 21074376

[B112] O’Hara C. M. Brenner F. W. Miller J. M. (2000). Classification, identification, and clinical significance of *Proteus*, *Providencia*, and *Morganella*. *Clin. Microbiol. Rev.* 13 534–546. 11023955 10.1128/cmr.13.4.534-546.2000 PMC88947

[B113] Ohno Y. Nakamura A. Hashimoto E. Matsutani H. Abe N. Fukuda S. (2017). Molecular epidemiology of carbapenemase-producing *Enterobacteriaceae* in a primary care hospital in Japan, 2010-2013. *J. Infect. Chemother.* 23 224–229. 10.1016/j.jiac.2016.12.013 28161293

[B114] Osano E. Arakawa Y. Wacharotayankun R. Ohta M. Horii T. Ito H. (1994). Molecular characterization of an enterobacterial metallo beta-lactamase found in a clinical isolate of *Serratia marcescens* that shows imipenem resistance. *Antimicrob. Agents Chemother.* 38 71–78. 10.1128/aac.38.1.71 8141584 PMC284399

[B115] Österblad M. Karah N. Halkilahti J. Sarkkinen H. Uhlin B. E. Jalava J. (2016). Rare detection of the *Acinetobacter* class D carbapenemase *bla*OXA-23 gene in *Proteus mirabilis*. *Antimicrob. Agents Chemother.* 60 3243–3245. 26902769 10.1128/AAC.03119-15 PMC4862524

[B116] Pagani L. Dell’Amico E. Migliavacca R. D’Andrea M. M. Giacobone E. Amicosante G. (2003). Multiple CTX-M-type extended-spectrum beta-lactamases in nosocomial isolates of *Enterobacteriaceae* from a hospital in northern Italy. *J. Clin. Microbiol.* 41 4264–4269. 10.1128/jcm.41.9.4264-4269.2003 12958255 PMC193787

[B117] Papagiannitsis C. C. Miriagou V. Kotsakis S. D. Tzelepi E. Vatopoulos A. C. Petinaki E. (2012). Characterization of a transmissible plasmid encoding VEB-1 and VIM-1 in *Proteus mirabilis*. *Antimicrob. Agents Chemother.* 56 4024–4025. 10.1128/aac.00470-12 22547621 PMC3393451

[B118] Papp-Wallace K. M. Mallo S. Bethel C. R. Taracila M. A. Hujer A. M. Fernández A. (2014). A kinetic analysis of the inhibition of FOX-4 β-lactamase, a plasmid-mediated AmpC cephalosporinase, by monocyclic β-lactams and carbapenems. *J. Antimicrob. Chemother.* 69 682–690. 10.1093/jac/dkt434 24235094 PMC3922156

[B119] Partridge S. R. Tsafnat G. Coiera E. Iredell J. R. (2009). Gene cassettes and cassette arrays in mobile resistance integrons. *FEMS Microbiol. Rev.* 33 757–784. 10.1111/j.1574-6976.2009.00175.x 19416365

[B120] Paton R. Miles R. S. Hood J. Amyes S. G. Miles R. S. Amyes S. G. (1993). ARI 1: beta-lactamase-mediated imipenem resistance in *Acinetobacter baumannii*. *Int. J. Antimicrob. Agents* 2 81–87. 10.1016/0924-8579(93)90045-7 18611526

[B121] Pavez M. Troncoso C. Osses I. Salazar R. Illesca V. Reydet P. (2019). High prevalence of CTX-M-1 group in ESBL-producing *Enterobacteriaceae* infection in intensive care units in southern Chile. *Braz. J. Infect. Dis.* 23 102–110. 10.1016/j.bjid.2019.03.002 31028724 PMC9425662

[B122] Perilli M. Dell’Amico E. Segatore B. de Massis M. R. Bianchi C. Luzzaro F. (2002). Molecular characterization of extended-spectrum β-lactamases produced by nosocomial isolates of *Enterobacteriaceae* from an Italian nationwide survey. *J. Clin. Microbiol.* 40 611–614. 10.1128/jcm.40.2.611-614.2002 11825979 PMC153390

[B123] Pfeifer Y. Trifonova A. Pietsch M. Brunner M. Todorova I. Gergova I. (2017). Clonal transmission of Gram-negative bacteria with carbapenemases NDM-1, VIM-1, and OXA-23/72 in a Bulgarian hospital. *Microb. Drug Resist.* 23 301–307. 10.1089/mdr.2016.0059 27459019

[B124] Philippon A. Slama P. Dény P. Labia R. (2016). A structure based classification of class A β-lactamases, a broadly diverse family of enzymes. *Clin. Microbiol. Rev.* 29 29–57. 10.1128/cmr.00019-15 26511485 PMC4771212

[B125] Poirel L. Bonnin R. A. Nordmann P. (2012). Genetic features of the widespread plasmid coding for the carbapenemase OXA-48. *Antimicrob. Agents Chemother.* 56 559–562. 10.1128/AAC.05289-11 22083465 PMC3256075

[B126] Poirel L. Dortet L. Bernabeu S. Nordmann P. (2011). Genetic features of *bla*NDM-1-positive *Enterobacteriaceae*. *Antimicrob. Agents Chemother.* 55 5403–5407. 10.1128/AAC.00585-11 21859933 PMC3195013

[B127] Poirel L. Figueiredo S. Cattoir V. Carattoli A. Nordmann P. (2008). *Acinetobacter radioresistens* as a silent source of carbapenem resistance for *Acinetobacter* spp. *Antimicrob. Agents Chemother.* 52 1252–1256. 10.1128/AAC.01304-07 18195058 PMC2292503

[B128] Poirel L. Héritier C. Tolün V. Nordmann P. (2004). Emergence of oxacillinase-mediated resistance to imipenem in *Klebsiella pneumoniae*. *Antimicrob. Agents Chemother.* 48 15–22. 10.1128/aac.48.1.15-22.2004 14693513 PMC310167

[B129] Poirel L. Naas T. Guibert M. Chaibi E. B. Labia R. Nordmann P. (1999). Molecular and biochemical characterization of VEB-1, a novel class A extended-spectrum beta-lactamase encoded by an *Escherichia coli* integron gene. *Antimicrob. Agents Chemother.* 43 573–581. 10.1128/aac.43.3.573 10049269 PMC89162

[B130] Poirel L. Rodriguez-Martinez J.-M. Mammeri H. Liard A. Nordmann P. (2005). Origin of plasmid-mediated quinolone resistance determinant QnrA. *Antimicrob. Agents Chemother.* 49 3523–3525. 10.1128/aac.49.8.3523-3525.2005 16048974 PMC1196254

[B131] Potron A. Hocquet D. Triponney P. Plésiat P. Bertrand X. Valot B. (2019). Carbapenem-susceptible OXA-23-producing *Proteus mirabilis* in the French community. *Antimicrob. Agents Chemother.* 63:e00191-19. 10.1128/AAC.00191-19 30962345 PMC6535531

[B132] Potron A. Poirel L. Elhag K. Al Yaqoubi F. Nordmann P. (2009). VEB-6 extended-spectrum beta-lactamase-producing *Proteus mirabilis* from Sultanate of Oman. *Int. J. Antimicrob. Agents* 34 493–494. 10.1016/j.ijantimicag.2009.05.002 19525094

[B133] Power P. Di Conza J. Rodríguez M. M. Ghiglione B. Ayala J. A. Casellas J. M. (2007). Biochemical characterization of PER-2 and genetic environment of *bla*PER-2. *Antimicrob. Agents Chemother.* 51 2359–2365. 17438050 10.1128/AAC.01395-06 PMC1913245

[B134] Qin S. Qi H. Zhang Q. Zhao D. Liu Z.-Z. Tian H. (2015). Emergence of extensively drug-resistant *Proteus mirabilis* harboring a conjugative NDM-1 plasmid and a novel *Salmonella* genomic island 1 variant, SGI1-Z. *Antimicrob. Agents Chemother.* 59 6601–6604. 10.1128/AAC.00292-15 26195511 PMC4576069

[B135] Quinteros M. Radice M. Gardella N. Rodriguez M. M. Costa N. Korbenfeld D. (2003). Extended-spectrum beta-lactamases in *Enterobacteriaceae* in Buenos Aires, Argentina, public hospitals. *Antimicrob. Agents Chemother.* 47 2864–2867. 10.1128/aac.47.9.2864-2867.2003 12936986 PMC182650

[B136] Raimondi A. Traverso A. Nikaido H. (1991). Imipenem- and meropenem-resistant mutants of *Enterobacter cloacae* and *Proteus rettgeri* lack porins. *Antimicrob. Agents Chemother.* 35 1174–1180. 10.1128/aac.35.6.1174 1656855 PMC284306

[B137] Ramos A. C. Cayô R. Carvalhaes C. G. Jové T. da Silva G. P. Sancho F. M. P. (2017). Dissemination of multidrug-resistant *Proteus mirabilis* clones carrying a novel integron-borne *bla*IMP-1 in a tertiary hospital. *Antimicrob. Agents Chemother.* 62:e01321-17. 10.1128/AAC.01321-17 29158274 PMC5786801

[B138] Rhimi-Mahjoubi F. Bernier M. Arlet G. Jemaa Z. B. Jouve P. Hammami A. (2002). Identification of plasmid-encoded cephalosporinase ACC-1 among various enterobacteria (*Klebsiella pneumoniae*, *Proteus mirabilis*, *Salmonella*) isolated from a Tunisian hospital (Sfax 997-2000). *Pathol. Biol.* 50 7–11. 10.1016/s0369-8114(01)00260-7 11873633

[B139] Robledo I. E. Aquino E. E. Santé M. I. Santana J. L. Otero D. M. León C. F. (2010). Detection of KPC in *Acinetobacter* spp. in Puerto Rico. *Antimicrob. Agents Chemother.* 54 1354–1357. 10.1128/AAC.00899-09 20038618 PMC2825984

[B140] Ryan M. P. Armshaw P. O’Halloran J. A. Pembroke J. T. (2017). Analysis and comparative genomics of R997, the first SXT/R391 integrative and conjugative element (ICE) of the Indian sub-continent. *Sci. Rep.* 7:8562. 10.1038/s41598-017-08735-y 28819148 PMC5561048

[B141] Sardelić S. Bedenić B. Sijak D. Colinon C. Kalenić S. (2010). Emergence of *Proteus mirabilis* isolates producing TEM-52 extended-spectrum beta-lactamases in Croatia. *Chemotherapy* 56 208–213. 10.1159/000316332 20551637

[B142] Saurina G. Quale J. M. Manikal V. M. Oydna E. Landman D. (2000). Antimicrobial resistance in *Enterobacteriaceae* in Brooklyn, NY: epidemiology and relation to antibiotic usage patterns. *J. Antimicrob. Chemother.* 45 895–898. 10.1093/jac/45.6.895 10837447

[B143] Schaffer J. N. Pearson M. M. (2015). *Proteus mirabilis* and urinary tract infections. *Microbiol. Spectr.* 3:10.1128/microbiolspec.UTI-0017-2013. 10.1128/microbiolspec.UTI-0017-2013 PMC4638163 26542036

[B144] Schmidt H. Hensel M. (2004). Pathogenicity islands in bacterial pathogenesis. *Clin. Microbiol. Rev.* 17 14–56. 10.1128/cmr.17.1.14-56.2004 14726454 PMC321463

[B145] Schultz E. Cloeckaert A. Doublet B. Madec J.-Y. Haenni M. (2017). Detection of SGI1/PGI1 elements and resistance to extended-spectrum cephalosporins in *Proteae* of animal origin in France. *Front. Microbiol.* 8:32. 10.3389/fmicb.2017.00032 28154560 PMC5243843

[B146] Schultz E. Haenni M. Mereghetti L. Siebor E. Neuwirth C. Madec J.-Y. (2015). Survey of multidrug resistance integrative mobilizable elements SGI1 and PGI1 in *Proteus mirabilis* in humans and dogs in France, 2010-13. *J. Antimicrob. Chemother.* 70 2543–2546. 10.1093/jac/dkv154 26066582

[B147] Seiffert S. N. Tinguely R. Lupo A. Neuwirth C. Perreten V. Endimiani A. (2013). High prevalence of extended-spectrum-cephalosporin-resistant *Enterobacteriaceae* in poultry meat in Switzerland: emergence of CMY-2- and VEB-6-possessing *Proteus mirabilis*. *Antimicrob. Agents Chemother.* 57 6406–6408. 10.1128/aac.01773-13 24080656 PMC3837880

[B148] Shen P. Wei Z. Jiang Y. Du X. Ji S. Yu Y. (2009). Novel genetic environment of the carbapenem-hydrolyzing beta-lactamase KPC-2 among *Enterobacteriaceae* in China. *Antimicrob. Agents Chemother.* 53 4333–4338. 10.1128/AAC.00260-09 19620332 PMC2764158

[B149] Sheng Z. Li J. Sheng G. Sheng J. Li L. (2010). Emergence of *Klebsiella pneumoniae* carbapenemase-producing *Proteus mirabilis* in Hangzhou, China. *Chin. Med. J.* 123 2568–2570. 21034629

[B150] Siebor E. Neuwirth C. (2011). The new variant of *Salmonella* genomic island 1 (SGI1-V) from a *Proteus mirabilis* French clinical isolate harbours *bla*VEB-6 and *qnrA1* in the multiple antibiotic resistance region. *J. Antimicrob. Chemother.* 66 2513–2520. 10.1093/jac/dkr335 21846670

[B151] Song W. Kim J. Bae I. K. Jeong S. H. Seo Y. H. Shin J. H. (2011). Chromosome-encoded AmpC and CTX-M extended-spectrum β-lactamases in clinical isolates of *Proteus mirabilis* from Korea. *Antimicrob. Agents Chemother.* 55 1414–1419. 10.1128/AAC.01835-09 21282448 PMC3067170

[B152] Spanu T. Luzzaro F. Perilli M. Amicosante G. Toniolo A. Fadda G. (2002). Occurrence of extended-spectrum beta-lactamases in members of the family *Enterobacteriaceae* in Italy: implications for resistance to beta-lactams and other antimicrobial drugs. *Antimicrob. Agents Chemother.* 46 196–202. 10.1128/aac.46.1.196-202.2002 11751134 PMC126983

[B153] Stock I. (2003). Natural antibiotic susceptibility of *Proteus* spp., with special reference to *P. mirabilis* and *P. penneri* strains. *J. Chemother.* 15 12–26. 12678409 10.1179/joc.2003.15.1.12

[B154] Takahashi I. Tsukamoto K. Harada M. Sawai T. (1983). Carbenicillin-hydrolyzing penicillinases of *Proteus mirabilis* and the PSE-type penicillinases of *Pseudomonas aeruginosa*. *Microbiol. Immunol.* 27 995–1004. 10.1111/j.1348-0421.1983.tb02934.x 6427559

[B155] Tamma P. D. Sharara S. L. Pana Z. D. Amoah J. Fisher S. L. Tekle T. (2019). Molecular epidemiology of ceftriaxone-nonsusceptible *Enterobacterales* isolates in an academic medical center in the United States. *Open Forum Infect. Dis.* 6:ofz353. 10.1093/ofid/ofz353 PMC6736082 31401649

[B156] Thomson K. S. Weber D. A. Sanders C. C. Sanders W. E. (1990). Beta-lactamase production in members of the family *Enterobacteriaceae* and resistance to beta-lactam-enzyme inhibitor combinations. *Antimicrob. Agents Chemother.* 34 622–627. 10.1128/aac.34.4.622 2344169 PMC171654

[B157] Tibbetts R. Frye J. G. Marschall J. Warren D. Dunne W. (2008). Detection of KPC-2 in a clinical isolate of *Proteus mirabilis* and first reported description of carbapenemase resistance caused by a KPC beta-lactamase in *P. mirabilis*. *J. Clin. Microbiol.* 46 3080–3083. 10.1128/JCM.00979-08 18632900 PMC2546724

[B158] Tonkic M. Mohar B. Sisko-Kraljević K. Mesko-Meglic K. Goić-Barisić I. Novak A. (2010). High prevalence and molecular characterization of extended-spectrum β-lactamase-producing *Proteus mirabilis* strains in southern Croatia. *J. Med. Microbiol.* 59 1185–1190. 10.1099/jmm.0.016964-0 20558587

[B159] Tran H. H. Ehsani S. Shibayama K. Matsui M. Suzuki S. Nguyen M. B. (2015). Common isolation of New Delhi metallo-beta-lactamase 1-producing *Enterobacteriaceae* in a large surgical hospital in Vietnam. *Eur. J. Clin. Microbiol. Infect. Dis.* 34 1247–1254. 10.1007/s10096-015-2345-6 25732142 PMC4426131

[B160] Tsai Y.-L. Wang M.-C. Hsueh P.-R. Liu M.-C. Hu R.-M. Wu Y.-J. (2015). Overexpression of an outer membrane protein associated with decreased susceptibility to carbapenems in *Proteus mirabilis*. *PLoS One* 10:e0120395. 10.1371/journal.pone.0120395 25756370 PMC4355480

[B161] Tsakris A. Ikonomidis A. Poulou A. Spanakis N. Pournaras S. Markou F. (2007). Transmission in the community of clonal *Proteus mirabilis* carrying VIM-1 metallo-beta-lactamase. *J. Antimicrob. Chemother.* 60 136–139. 10.1093/jac/dkm138 17491004

[B162] van Dijk K. Voets G. M. Scharringa J. Voskuil S. Fluit A. C. Rottier W. C. (2014). A disc diffusion assay for detection of class A, B and OXA-48 carbapenemases in *Enterobacteriaceae* using phenyl boronic acid, dipicolinic acid and temocillin. *Clin. Microbiol. Infect.* 20 345–349. 10.1111/1469-0691.12322 23927659

[B163] Verdet C. Arlet G. Barnaud G. Lagrange P. H. Philippon A. (2000). A novel integron in *Salmonella enterica* serovar enteritidis, carrying the *bla*DHA-1 gene and its regulator gene *ampR*, originated from *Morganella morganii*. *Antimicrob. Agents Chemother.* 44 222–225. 10.1128/aac.44.1.222-225.2000 10602756 PMC89661

[B164] Verdet C. Arlet G. Ben Redjeb S. Ben Hassen A. Lagrange P. H. Philippon A. (1998). Characterisation of CMY-4, an AmpC-type plasmid-mediated beta-lactamase in a Tunisian clinical isolate of *Proteus mirabilis*. *FEMS Microbiol. Lett.* 169 235–240. 10.1016/s0378-1097(98)00491-1 9868767

[B165] Verdet C. Gautier V. Chachaty E. Ronco E. Hidri N. Decré D. (2009). Genetic context of plasmid-carried *bla*CMY-2-like genes in *Enterobacteriaceae*. *Antimicrob. Agents Chemother.* 53 4002–4006. 10.1128/AAC.00753-08 19596889 PMC2737857

[B166] Villar H. (1997). Permeability to carbapenems of *Proteus mirabilis* mutants selected for resistance to imipenem or other beta-lactams. *J. Antimicrob. Chemother.* 40 365–370. 10.1093/jac/40.3.365 9338488

[B167] Vourli S. Tsorlini H. Katsifa H. Polemis M. Tzouvelekis L. S. Kontodimou A. (2006). Emergence of *Proteus mirabilis* carrying the *bla* metallo-beta-lactamase gene. *Clin. Microbiol. Infect.* 12 691–694. 10.1111/j.1469-0691.2006.01489.x 16774572

[B168] Wachino J.-I. Yamane K. Shibayama K. Kurokawa H. Shibata N. Suzuki S. (2006). Novel plasmid-mediated 16S rRNA methylase, RmtC, found in a *Proteus mirabilis* isolate demonstrating extraordinary high-level resistance against various aminoglycosides. *Antimicrob. Agents Chemother.* 50 178–184. 10.1128/aac.50.1.178-184.2006 16377684 PMC1346777

[B169] Wang M. Guo Q. Xu X. Wang X. Ye X. Wu S. (2009). New plasmid-mediated quinolone resistance gene, *qnrC*, found in a clinical isolate of *Proteus mirabilis*. *Antimicrob. Agents Chemother.* 53 1892–1897. 10.1128/AAC.01400-08 19258263 PMC2681562

[B170] Watanabe M. Iyobe S. Inoue M. Mitsuhashi S. (1991). Transferable imipenem resistance in *Pseudomonas aeruginosa*. *Antimicrob. Agents Chemother.* 35 147–151. 10.1128/aac.35.1.147 1901695 PMC244956

[B171] Williamson D. A. Sidjabat H. E. Freeman J. T. Roberts S. A. Silvey A. Woodhouse R. (2012). Identification and molecular characterisation of New Delhi metallo-β-lactamase-1 (NDM-1)- and NDM-6-producing *Enterobacteriaceae* from New Zealand hospitals. *Int. J. Antimicrob. Agents* 39 529–533. 10.1016/j.ijantimicag.2012.02.017 22526013

[B172] Wolter D. J. Kurpiel P. M. Woodford N. Palepou M.-F. I. Goering R. V. Hanson N. D. (2009). Phenotypic and enzymatic comparative analysis of the novel KPC variant KPC-5 and its evolutionary variants, KPC-2 and KPC-4. *Antimicrob. Agents Chemother.* 53 557–562. 10.1128/AAC.00734-08 19015357 PMC2630594

[B173] Yan J.-J. Ko W.-C. Jung Y.-C. Chuang C.-L. Wu J.-J. (2002). Emergence of *Klebsiella pneumoniae* isolates producing inducible DHA-1 beta-lactamase in a university hospital in Taiwan. *J. Clin. Microbiol.* 40 3121–3126. 10.1128/jcm.40.9.3121-3126.2002 12202541 PMC130748

[B174] Yigit H. Queenan A. M. Anderson G. J. Domenech-Sanchez A. Biddle J. W. Steward C. D. (2001). Novel carbapenem-hydrolyzing beta-lactamase, KPC-1, from a carbapenem-resistant strain of *Klebsiella pneumoniae*. *Antimicrob. Agents Chemother.* 45 1151–1161. 10.1128/aac.45.4.1151-1161.2001 11257029 PMC90438

[B175] Yokoyama K. Doi Y. Yamane K. Kurokawa H. Shibata N. Shibayama K. (2003). Acquisition of 16S rRNA methylase gene in *Pseudomonas aeruginosa*. *Lancet* 362 1888–1893. 10.1016/s0140-6736(03)14959-8 14667745

[B176] Yong D. Lim Y. Song W. Choi Y. S. Park D.-Y. Lee H. (2005). Plasmid-mediated, inducible AmpC beta-lactamase (DHA-1)-producing *Enterobacteriaceae* at a Korean hospital: wide dissemination in *Klebsiella pneumoniae* and *Klebsiella oxytoca* and emergence in *Proteus mirabilis*. *Diagn. Microbiol. Infect. Dis.* 53 65–70. 10.1016/j.diagmicrobio.2005.03.008 15936167

[B177] Yong D. Lim Y. S. Roh K. H. Choi Y. S. Park D.-Y. Yum J. H. (2006). The first detection of CTX-M-14 extended-spectrum β-lactamase among diverse β-lactamase–producing *Proteus mirabilis* clinical isolates. *Diagn. Microbiol. Infect. Dis.* 54 237–239. 10.1016/j.diagmicrobio.2005.09.013 16427241

[B178] Yong D. Toleman M. A. Giske C. G. Cho H. S. Sundman K. Lee K. (2009). Characterization of a new metallo-beta-lactamase gene, *bla*NDM-1, and a novel erythromycin esterase gene carried on a unique genetic structure in *Klebsiella pneumoniae* sequence type 14 from India. *Antimicrob. Agents Chemother.* 53 5046–5054. 10.1128/aac.00774-09 19770275 PMC2786356

[B179] Zabel M. D. Bunch P. K. Clark D. P. (2000). Regulation of the *thdF* gene, which is involved in thiophene oxidation by *Escherichia coli* K-12. *Microbios* 101 89–103. 10738982

[B180] Zhang F. Xie L. Wang X. Han L. Guo X. Ni Y. (2016). Further spread of *bla*NDM-5 in *Enterobacteriaceae* via IncX3 plasmids in Shanghai, China. *Front. Microbiol.* 7:424. 10.3389/fmicb.2016.00424 27065982 PMC4811927

[B181] Zhang S. Sun J. Liao X.-P. Hu Q.-J. Liu B.-T. Fang L.-X. (2013). Prevalence and plasmid characterization of the *qnrD* determinant in *Enterobacteriaceae* isolated from animals, retail meat products, and humans. *Microb. Drug Resist.* 19 331–335. 10.1089/mdr.2012.0146 23557071

[B182] Zong Z. Partridge S. R. Iredell J. R. (2009). A *bla*VEB-1 variant, *bla*VEB-6, associated with repeated elements in a complex genetic structure. *Antimicrob. Agents Chemother.* 53 1693–1697. 10.1128/AAC.01313-08 19139283 PMC2663121

